# The role of benthic macrofauna in the trophic transfer of mercury in a low-diversity temperate coastal ecosystem (Puck Lagoon, southern Baltic Sea)

**DOI:** 10.1007/s10661-019-7257-y

**Published:** 2019-02-07

**Authors:** Agnieszka Jędruch, Magdalena Bełdowska, Marcelina Ziółkowska

**Affiliations:** Institute of Oceanography, University of Gdańsk, Piłsudskiego 46, 81-378 Gdynia, Poland

**Keywords:** Mercury, Macrozoobenthos, Coastal food web, Trophic transfer, Biomagnification, Baltic Sea

## Abstract

Mercury (Hg) is a global pollutant that affects human and ecosystem health. Hg is a serious threat especially for the marine environment, in which it undergoes bioaccumulation and biomagnification, reaching elevated concentrations in fish and other seafood. The research aimed at investigating the trophodynamics of Hg in the basal links of the marine food chain: benthic macrofauna and its main food sources (i.e. suspended and sediment organic matter, micro- and macrophytobenthos). The results showed that both the amount and the origin of organic matter affected the Hg level in particular trophic groups of macrozoobenthos. The intensive inflow of terrestrial material influenced the enrichment of suspended particles and microphytobenthos in Hg, leading to increased Hg concentrations in filter-feeding macrofauna. The input of Hg-rich marine matter transported from the deeper parts of the Gulf of Gdańsk along with the near-bottom currents caused higher Hg levels in deposit feeders. The biomagnification factor (BMF) of Hg through benthic food web was dependent on environmental conditions occurring in the studied areas, in particular, factors favouring the growth and fecundity of macrofauna. Consequently, as a result of biodilution, the trophic transfer of Hg was less effective in a more productive region, despite the elevated Hg concentrations in dietary components of the macrofauna and in the surrounding environment.

**Electronic supplementary material:**

The online version of this article (10.1007/s10661-019-7257-y) contains supplementary material, which is available to authorized users.

## Introduction

Mercury (Hg) is considered to be one of the most dangerous global environmental pollutants and has been the subject of numerous scientific studies over many years. This is primarily related to the chemical and biological activity of Hg, its high mobility, its rapid spread in the environment and its bioaccumulation and biomagnification abilities (Frörstner and Wittman 1981; Jackson 1998).

The Baltic, as a semi-enclosed sea with limited water exchange, surrounded by industrialised areas, has been the scene of uncontrolled discharge of contaminants containing Hg for decades. This has resulted in elevated concentrations of Hg the water and sediments and also in the organisms that inhabit the Baltic Sea (Wrembel 1993). Reductions in the Hg load entering the sea have only been observed since the 1990s (HELCOM 2010; Bełdowska 2016). In the case of the marine environment, this period is too short to show a significant reduction in the Hg concentration in its components (in other words, to get the ecosystem response to the introduced emission restrictions). This is shown by studies on Hg concentration in sediments (Bełdowska et al. 2013; Jędruch et al. 2015) or organisms that are associated with the marine bottom (Bełdowska et al. 2015, 2016b). These earlier studies also focused on the determination of Hg sources and loads entering the Baltic Sea. It is well known that Hg is mainly introduced into the Baltic via the inflow of river water and atmospheric deposition (HELCOM 2010; Bełdowska et al. 2015). Our results show that in the case of coastal areas, the Hg leaching from the land (i.e. due to coastal erosion and surface runoff from urbanised areas) plays an important role in the Hg load reaching the marine environment (Bełdowska et al. 2016a; Jędruch et al. 2017; Kwasigroch et al. 2018). Such sources are rarely considered when assessing the Hg budget in the marine environment, although in regions with a high inflow of terrestrial matter, a significant proportion of Hg accumulates in the coastal zone, especially in bottom sediments, from where it can enter the trophic chain by both phyto- and zoobenthic organisms (Bełdowska et al. 2015, 2016b; Jędruch et al. 2017, 2018a).

The evolutionary young age of the Baltic Sea in combination with the predominant brackish conditions results in naturally low species diversity, facilitating analyses of environmental response to anthropogenic pressures (Reusch et al. 2018). Baltic populations can serve as a test case on how trophodynamics of Hg may be influenced by global changes in environmental conditions observed in recent years (e.g. warming, acidification, oxygen depletion). Previous works shows that the physico-chemical parameters of water and its productivity play an important role in Hg bioaccumulation and tropic transfer, especially in low- and medium-trophic level consumers (Bełdowska et al. 2016b; Chouvelon et al. 2018; Jędruch et al. 2018a). Additionally, the species-poor ecosystem of the Baltic Sea and its short postglacial history results in a relatively simple food web structure (Nordstöm et al. 2009; Sokołowski et al. 2012) and contributes to the increased trophic transfer of Hg (Lavoie et al. 2013).

Benthic organisms are an important element of the marine ecosystem—as a primary link in the trophic chain, they exist as a significant dietary component for many fish, seabirds, and humans. The main conditions that determine which fauna settle on the bottom are salinity, temperature, type of substrate (soft or hard bottom), the concentration of dissolved oxygen and the availability of food. The highest density and the most diverse composition of zoobenthic species are found in coastal waters. This is associated primarily with the varied topography, diverse habitat and higher productivity of shallow areas (Szymelfenig 2008). The type and abundance of zoobenthos are also influenced by the presence of underwater meadows—here, the higher density of phytobenthos creates favourable conditions for bottom-dwelling fauna (Jankowska et al. 2014; Sokołowski et al. 2015). Coastal ecosystem, are also characterised by functional richness of macrofauna, compared to open areas (Törnroos and Bonsdorff 2012). Even in the relatively poor in species Puck Lagoon, the benthic organisms have diversified functional properties (related to morphological, physiological, or behavioural aspects) and consequently varied feeding habits (e.g. consumption of suspended or sediment organic matter, plankton, microphytobenthos, live and dead animal tissues) (Jankowska et al. 2018). Thus, bottom fauna can accumulate Hg originating from many sources.

The transfer of Hg in the aquatic trophic chain has been the subject of many studies in recent years, with most of these studies focusing on freshwater bodies (e.g. Campbell et al. 2003; Wyn et al. 2009; Lavoie et al. 2010; Molina et al. 2010; Kidd et al. 2012). However, studies have shown that the process of bioaccumulation and biomagnification is more efficient in the marine environment than in the freshwater environment (Lavoie et al. 2013; Riyadi et al. 2015; Bełdowska and Falkowska 2016). A compilation of data on the transfer of Hg in the trophic chain in more than 200 aqueous systems indicates that many papers contain critical gaps in data and methodology (Lavoie et al. 2013). In many cases, the study was conducted only on a selected group of organisms (e.g. zooplankton, molluscs) or those of a high trophic status (e.g. predatory fish, birds). Neither the sources of Hg nor the concentration in the surrounding environment was considered. Furthermore, the research material was often collected only in one season (usually summer), thereby failing to take into account differences in the metabolic processes of organisms and the physico-chemical parameters of the environment, as well as changes in the amounts of autochthonous and allochthonous organic matter and Hg. Importantly, the role of the organisms from the lowest trophic levels in the trophodynamics of Hg in marine food chain is still not well recognised and requires further investigation (Chouvelon et al. 2018).

The purpose of this paper was, therefore, to determine the role of macrofauna in the transfer of Hg to the food chain at the sediment-primary production interface in a low-diversity temperate ecosystem, taking into account sources of Hg and the position of organisms in the trophic web. The authors decided to investigate the total Hg level in the most important elements of the benthic environment of the coastal area, which is associated with the fact that, as shown by previous studies, almost all Hg (94–99%) accumulated in the macrozoobenthos occurs in bioavailable, labile forms (Jędruch et al. 2018b).

## Materials and methods

### **Study area**

**Fig. 1:**
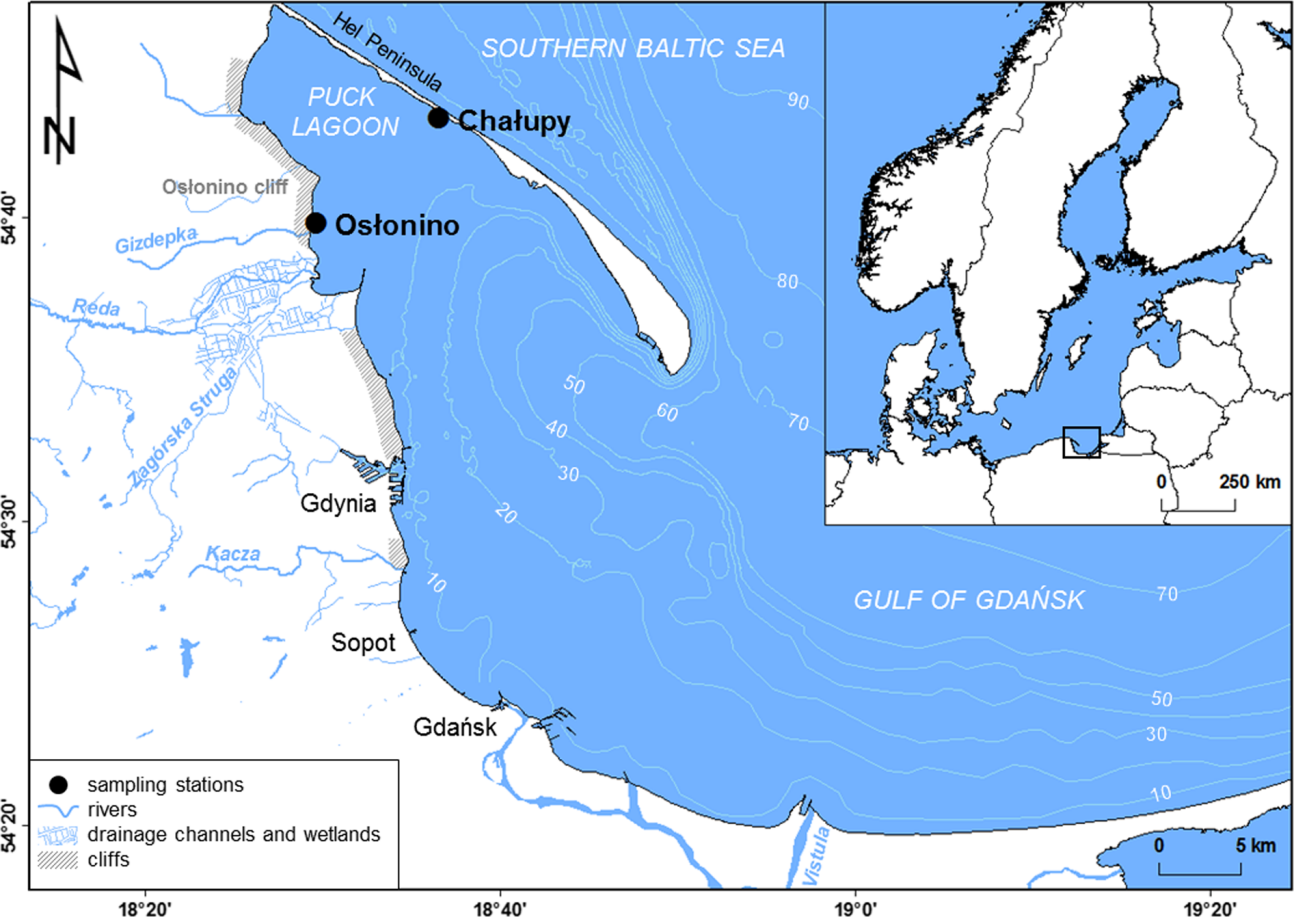
Sampling stations in the Puck Lagoon (southern Baltic Sea)

The research was carried out at two sites in the Gulf of Gdańsk located in the southeastern part of the Baltic Sea (Fig. 1). The Gulf of Gdańsk area abounds in anthropogenic pollution sources, and most of these are located within the Tricity agglomeration situated on the southwestern coast (numerous facilities related to shipbuilding, energy and petrochemical industry and tourism). The main river entering this basin is the Vistula, the second largest river flowing into the Baltic. Stations in Osłonino and Chałupy were located in the coastal zone (depth 1 m) of the Gulf of Gdańsk, in an area sheltered by the Hel Peninsula—the Puck Lagoon. For years, the Puck Lagoon has been exposed for an uncontrolled discharge of pollutants from the surrounding land. The accumulation of pollutants in the Puck Lagoon was favoured by shallow depth (average 3 m), the shape of the sea bottom, and limited water exchange. On the other hand, these conditions are conducive to the growth of aquatic organisms—the Puck Lagoon ecosystem possesses the greatest biodiversity of any area within the Polish coastal zone of the Baltic (the species richness is about two times greater than in the open part of the Gulf of Gdańsk) (Sokołowski 2009). It is related to the fact that much of the bottom of the Puck Lagoon is covered by underwater meadows, including the most important species of seagrass—*Zostera marina* (Węsławski et al. 2013; Jankowska et al. 2014).

The station located on the western shore of the Puck Lagoon (Osłonino) was characterised by a large inflow of terrigenous matter associated with the inflow of few small rivers and the erosion of cliffs located in the vicinity. The station situated in the northern part of the Puck Lagoon (Chałupy) was slightly influenced by land—the main sources of organic matter were primary production and marine material transported from deeper parts of the sea (Bełdowska et al. 2016a; Jędruch et al. 2017).

### **Sample collection**

Samples of benthic macrofauna and components of their diet were collected once a month from December 2011 to May 2013. The total number of sampling campaigns was 18; however, the biological material was collected 14 times at Osłonino station and 15 times at Chałupy station. During some winter months, samples of biota were not collected due to the presence of ice cover (at the Osłonino station: in February 2012 and from January 2013 to March 2013; at the Chałupy station: in February 2012 and from December 2012 to February 2013). Samples of macrofauna were collected using a manual Van Veen grab sampler with a grab area of 250 cm^2^, in three replicates. Collected surface sediments were then sieved through a 0.5-mm mesh. The live biological material was placed in containers with sea water in situ and transported to the laboratory. All samples were kept aerated until analysis. Additionally, on each occasion, samples of other ecosystem components of the near-bottom zone were collected: subsurface sea water (in order to filter out the suspended particulate matter (SPM), phyto- and zooplankton, benthic macrophytes together with epiphytes, microalgal biofilm from rocks (epilithon), surface sediments (the upper 10 cm), pore waters (using a rhizon in situ sampler) and suspended matter at the sediment–water interface (FLSM)). Sampling was carried out according to the commonly used methods described inter alia by Seeberg-Elverfeldt et al. (2005), Durai and Pandiyan (2011), Kelly and Zgrundo (2013) Bełdowska et al. (2015, 2016b), Bełdowska and Kobos (2016), Bełdowska and Mudrak-Cegiołka (2017) and Jędruch et al. (2017). In cases where they were observed in the area of research stations, small benthic fish were also collected (as potential consumers of macrozoobenthos). Additionally, the basic parameters (temperature, salinity and pH) of near-bottom water were measured using the multi-parameter portable metre (ProfiLine Multi 3320, WTW, Germany). The redox potential was measured using an Eh-metre (Microscale Measurements, model MB II mV) (Graca et al. 2016). Immediately upon arrival at the laboratory, subsamples of the surface sediments were collected in order to determine their basic properties: water content (*W*), organic matter content estimated by loss on ignition (LOI) at 550 °C (Santisteban et al. 2004) and grain-size distribution in accordance with commonly used methods described in an earlier study by Jędruch et al. (2015).

### **Biological analysis**

Laboratory analysis of macrozoobenthos samples included the determination of species composition, abundance and biomass. Taxonomic identification of organisms was carried out on the basis of observations of their morphological features and available taxonomic keys (Żmudziński 1990; Barnes 1994). Systematisation and nomenclature of macrofauna organisms were adopted in accordance with the World Register of Marine Species (WoRMS, www.marinespecies.org). To determine the biomass (g m^2^) of the organisms, they were freeze dried. The obtained results of abundance and biomass were converted to indicate 1 m^2^ of the bottom surface. The analysis of all macrozoobenthos samples was carried out in accordance with standards applicable in biological laboratory testing procedures used as part of an international monitoring of the Baltic Sea (HELCOM 1988).

For samples from the coastal zone, the following common community indices were used to estimate the structure of the macrozoobenthos—the dominance index (*D*) and the frequency of occurrence (C) (Tischler 1949; Trojan 1980). The dominance index of each taxon was calculated according to the formula:

1 $$ D=\frac{S_{\mathrm{taxa}}}{S}\bullet 100 $$

where *S*_taxa_ is the sum of the abundance (or biomass) of individuals belonging to a particular taxon in all examined samples and *S* is the sum of the abundance (or biomass) of all individuals in all samples. Eudominant taxa are defined as *S* ≥ 10%, taxa with 10 > *D* ≥ 5 are dominant, taxa with 5 > *D* ≥ 2 are subdominant, taxa with 2 > *D* ≥ 1 are recedent and taxa with *D* < 1 are classified as subrecedent (Trojan 1980). The frequency of occurrence, also referred to as a constancy index, revealed the dispersion of taxa in the investigated communities and was obtained from the following equation:

2 $$ C=\frac{n_{\mathrm{taxa}}}{N}\bullet 100 $$

where *n*_taxa_ is the number of samples containing a given taxon and *N* is the total number of samples. Euconstant taxa are defined as having *C* > 75%, taxa with 75% ≥ *C* > 50% are constant, taxa with 50% ≥ *C* > 25% are referred to as accessory taxa and taxa with *C* < 25% belong to the group of accidental taxa (Napiórkowska-Krzebietke 2009; Marković et al. 2015).

Benthic macroalgae and vascular plants were identified to the lowest possible taxonomic level within 2 days of collection using the taxonomy key given by Braune and Guiry (2011). Nomenclature followed Algae Base (www.algaebase.org) and the European Register of Marine Species (ERMS, www.marbef.org/data/erms.php). Fish were identified to the species according to the atlas by Heessen et al. (2015).

### **Determination of Hg concentration**

The preparation of macrozoobenthos samples for Hg analysis involved placing the taxonomically segregated biological material into single-use Eppendorf Safe-Lock Conical Tubes (volume 1.5 or 5.0 mL), which have been previously rinsed with 4 M HNO_3_, dried at 60 °C and weighed. In the cases of bivalves (*Cerastoderma glaucum*, *Limecola baltica*, *Mya arenaria*), gastropods (*Peringia* sp., *Radix labiata*, *Theodoxus fluviatilis*) and crab (*Rhithropanopeus harrisii*), the soft body was separated from the shell or carapace using the stainless-steel scalpel blade (only soft body parts have been subjected to the further Hg analysis). The number of organisms placed in one tube was dependent on the individual mass of individuals of a given taxon (i.e. in the case of relatively large bivalves ranged from a few to a dozen or so, while in the case of small organisms such as gastropods, it accounted for tens of individuals). In particular tube, only the organisms of the same species, collected on the same station and in the same month (during the same sampling campaign), were placed. The same applies to the composite samples (prepared in the case when the biomass of organisms from a given species was small)—combining the sample included only the merging of material collected at the same time during the three replications. Prior to analysis, all samples (previously stored at a temperature of − 20 °C) were freeze-dried (Alpha 1-4 LDplus, Martin Christ, Germany). Additionally, the samples of surface sediments were homogenised in a ball mill with agate vials (8000D Mixer/Mill, SPEX SamplePrep, USA). Total mercury concentrations (Hg_TOT_) in the collected material were determined using atomic absorption spectrometry (AAS) on the AMA-245 mercury analyser (Altec, Czech Republic). The analyses included the determination of Hg_TOT_ in each of the designated macrozoobenthos species, in one to five repetitions, depending on the mass of the organisms. Hg_TOT_ concentrations were also analysed in primary producers (epilithon, epiphyton, macroalgae, and vascular plants) and benthic fish, as well as in SPM, FLSM and surface sediments. These analyses were performed by triplicate in the case of biological material and with five repetitions in the case of surface sediments. The quality control of the method included the analysis of certified reference materials (BCR 414—plankton, BCR 279—macroalgae *Ulva lactuca*, GBW 07314—offshore sediment). The method was characterised by high recovery (96–98%), and the standard deviation did not exceed 5%. The limit of detection (LOD) was 0.01 ng g^−1^.

The concentrations of Hg_TOT_ measured in abiotic and biotic components were expressed in terms of dry weight (dw). In the case of surface sediments, concentrations of Hg_TOT_ were also presented as values normalised in relation to the percentage of fine-grained sediment fraction (Hg_FSF_) and the proportion of organic matter (Hg_LOI_) to compensate the differences in sediments parameters. The normalisation of Hg concentrations was performed in accordance with formulae presented in an earlier study by Jędruch et al. (2015):

3 $$ {\mathrm{Hg}}_{\mathrm{LOI}}=\frac{{\mathrm{Hg}}_{\mathrm{TOT}}}{\mathrm{LOI}\ \left({10}^{-2}\right)} $$

4 $$ {\mathrm{Hg}}_{\mathrm{FSF}}=\frac{{\mathrm{Hg}}_{\mathrm{TOT}}}{\mathrm{FSF}\ \left({10}^{-2}\right)} $$

where Hg_LOI_ and Hg_FSF_ are concentration of total Hg (Hg_TOT_) normalised to the content of organic matter (LOI) and fine sediment fraction (FSF) in the surface sediments, respectively.

### Determination of Hg bioaccumulation and biomagnification

To evaluate the ability of benthic organisms to accumulate Hg from surface sediment, the biota–sediment accumulation factor (BSAF) was calculated following the formula suggested by Szefer et al. (1999):

5 $$ \mathrm{BSAF}=\frac{{\mathrm{Hg}}_{\mathrm{biota}}}{{\mathrm{Hg}}_{\mathrm{sediment}}} $$

where Hg_biota_ and Hg_sediment_ are the concentrations of Hg in a given taxon and surface sediments, respectively.

In order to determine Hg accumulation in the trophic chain, the stable isotopic values of analysed components were used. The carbon and nitrogen isotopic compositions of suspended particulate matter (particulate organic matter, POM), sediments (sediment organic matter, SOM) and benthic organisms were expressed with the standard *δ* unit notation, given in parts per thousand (‰), according to the following equation:

6 $$ \delta X=\left[\left(\frac{R_{\mathrm{sample}}}{R_{\mathrm{standard}}}\right)-1\right]\bullet 1000 $$

where *X* is the stable isotope ratio of *δ*^13^C or *δ*^15^N, *R* is the ratio of ^13^C/^12^C for carbon or ^15^N/^14^N for nitrogen for sample and for standard reference material (Pee Dee Belemnite (PDB) for *δ*^13^C and air for *δ*^15^N) (Khan et al. 2015). Data pertaining to the *δ*^13^C and *δ*^15^N values in selected ecosystem components used in this study were taken from a simultaneous research project being conducted by the authors (Jędruch et al. 2017), as well as from literature (Hansson et al. 1997; Riera et al. 1999; Attrill et al. 2009; Nordstöm et al. 2009; Sokołowski 2009; Kolb et al. 2010; Riera 2010; Olsen et al. 2011; Prado et al. 2013; Karlson et al. 2015; Jankowska et al. 2016; Jankowska 2017; Jankowska et al. 2018). The majority of literature data were obtained from the Puck Lagoon and at similar time as this study (Table SI).

As nitrogen stable isotope (*δ*^15^N) values provide an indication of the trophic position of an organism in the food web, the trophic level (TL) for each group of primary producers and consumers was estimated, using the following equation (Hobson and Welch 1992):

7 $$ \mathrm{TL}=\frac{\delta^{15}{\mathrm{N}}_{\mathrm{consumer}}-{\delta}^{15}{\mathrm{N}}_{\mathrm{baseline}}}{\Delta ^{15}\mathrm{N}}+2 $$

where TL is the trophic level of a given consumer, and *δ*^15^N_consumer_ and *δ*^15^N_baseline_ are *δ*^15^N values of a consumer and the baseline organisms, respectively. The *δ*^15^N_baseline_ for the study area (8.4) was previously calculated by Sokołowski et al. (2012) for all primary consumers in the study area. The Δ^15^N is a trophic enrichment factor for *δ*^15^N (3.4‰)—the value by which the *δ*^15^N increases in the subsequent levels of the trophic chain (Fry 2006; Sokołowski 2009; Lavoie et al. 2013).

To estimate the biomagnification potential of Hg, also referred to as the trophic magnification slope (TMS), a theoretical model developed by Broman et al. (1992) and Rolff et al. (1993) for the trophic transfer of contaminants was applied:

8 $$ {\mathrm{Hg}}_{\mathrm{compartment}}={\mathrm{Ae}}^{\mathrm{B}{\delta}^{15}\mathrm{N}} $$

where Hg_compartment_ is the Hg_TOT_ concentration in sediments, particulate suspended matter and benthic organisms and A and B are the function parameters of Hg concentration and nitrogen isotopic value (*δ*^15^N). The model constant A is a scaling factor that depends on Hg concentration at the base of the food chain (Rolff et al. 1993). In order to estimate the parameters A and B, variables from Eq. 8 were converted using logarithmic transformation, resulting in linear regression:

9 $$ \ln {\mathrm{Hg}}_{\mathrm{compartment}}={B\delta}^{15}\mathrm{N}+\mathrm{lnA} $$

The slope of this regression (*B*) is the TMS (Broman et al. 1992), a parameter which is routinely used as an indicator of biomagnification potential of Hg in food webs around the world (i.e. Cai et al. 2007; Lavoie et al. 2010; Kim et al. 2012; Chouvelon et al. 2018). A positive slope (TMS > 0) indicates Hg biomagnification in a food web, while a negative one (TMS < 0) indicates trophic diminution of Hg (Lavoie et al. 2013; Riyadi et al. 2015). Assuming that a change of stable nitrogen isotope of 3.4‰ represents one trophic level in the studied area (Sokołowski 2009; Sokołowski et al. 2015), the biomagnification factor (BMF), representing the increase of Hg concentration per trophic level, was calculated on the basis of antilogarithmic back-transformation (Hobson and Welch 1992):

10 $$ \mathrm{BMF}={\mathrm{e}}^{3.4\mathrm{B}} $$

### **Processing results**

Statistical analysis of the obtained results was carried out using *STATISTICA 12* software (StatSoft). The hypotheses were tested at statistical significance level of *p* < 0.05. The analysed data were not characterised by the normal distribution (Shapiro–Wilk test, *p* = 0.00). In order to determine the significance of differences, the non-parametric *U* Mann–Whitney or Kruskal–Wallis’ tests were used, as well as the multiple comparison post hoc Dunn’s test. The relationships between the analysed variables were determined on the basis of the Spearman’s coefficient. The outliers were determined by multiplying the interquartile range (IQR) by 1.5, while the extreme values by multiplying IQR by 3 (Tukey 1997). The map of the study area with the distribution of sampling stations was created using *ArcMap 10.4* software (ESRI) with the WGS1984 geographic coordinate system and UTM zone 33N projection. The spatial data were provided courtesy of the GIS Centre, University of Gdańsk (www.ocean.ug.edu.pl/~oceju/CentrumGIS).

## Results

### **Characteristic of the benthic habitat**

The sampling stations located in the Puck Lagoon (Fig. 1) differed in terms of environmental conditions and type of bottom. The area of Osłonino was influenced by a strong inflow of suspended particulate matter (SPM), which was reflected by its high concentration in water at this station (median 36.1 mg L^−1^) (Table 1). In comparison, the concentration of SPM in Chałupy was three times lower (median 12.2 mg L^−1^). At both research stations, the surface sediments was sandy and medium-grained fraction of sands was dominant (grain diameter 250–500 μm). However, the sediments at Osłonino stations contained more finest sediment fraction (FSF) with grain diameter below 63 μm (median 1.6%) than sediments in Chałupy (median 0.3%) (Table 1). It was similar in the case of the content of organic matter, estimated by loss of ignition (LOI), which share in Osłonino was higher (median 1.0%) in comparison to Chałupy (median 0.3%). The surface sediments collected at Osłonino and Chałupy were characterised by different oxygen conditions. Values of oxidation–reduction potential (Eh) measured in the area of Osłonino were lower (median 235.0 mV) than in Chałupy (median 299.0 mV). What is more, the Eh measured in Osłonino varied in a lesser range (71.0–464.0 mV) in comparison to Chałupy (− 465.0–695.0 mV), during the study period. The other environmental parameters (salinity and pH), measured in the near-bottom water, were slightly lower in the area of Osłonino (median *S* 6.4 PSU; median pH 7.8) than in Chałupy (median *S* 6.5 PSU; median pH 8.0). However, at Osłonino station, these parameters periodically decreased and reached values as low as 4.3 PSU in the case of salinity, and 5.9 in the case of pH, while in Chałupy the measured values varied in a narrower range (Table 1). The sampling station also differed in terms of bottom vegetation. In the area of Chałupy, the density of macrophytobenthos was higher in comparison to Osłonino station. Differences in species composition of benthic plants were also observed. In Chałupy, the rich occurrence of vascular plants, such as seagrass (*Zostera marina*) and brackish pondweed (*Potamogeton* spp. *Zannichelia palustris*), was observed. Among the macroalgae, the sea lettuce *Enteromorpha* sp. and charophyte green algae (*Chara baltica*) dominated at the Chałupy station. At Osłonino, vascular plants were less numerous and were represented mainly by *Potamogeton* spp. Macroalgae were in turn dominated by filamentous green algae *Cladophora* sp. and brown algae *Pylaiella littoralis.*

**Table 1:** Basic parameters of the near-bottom zone at stations located in the Puck Lagoon (southern Baltic Sea) in years 2011–2013

Station	Basic statistics	SPM concentration	Surface sediment properties			Environmental parameters			
		(mg L^−1^)	*W* (%)	LOI (%)	FSF (%)	*T* (°C)	*S* (PSU)	pH	Eh (mV)
Osłonino	*N*	54	36	36	36	36	36	28	28
	Mean	44.8	17.9	1.0	1.9	10.2	5.8	7.7	256.6
	Median	36.1	17.3	1.0	1.6	9.5	*6.4*	7.8	235.0
	Min	8.0	15.5	0.2	0.2	− 0.1	4.3	5.9	71.0
	Max	140.6	22.1	2.0	6.5	24.0	7.1	8.1	464.0
Chałupy	*N*	51	36	36	36	36	36	30	30
	Mean	33.6	16.1	0.7	0.4	10.2	6.4	8.0	271.7
	Median	12.2	15.2	0.3	0.3	9.0	6.5	8.0	299.0
	Min	5.6	12.1	0.2	0.1	0.3	6.0	7.0	− 435.0
	Max	70.6	18.2	1.8	1.2	21.2	7.0	8.7	695.0

*N* number of analysed samples (including the number of repetitions: three in the case of suspended particulate matter, two in the case of sediment and environmental parameters), *SPM* suspended particulate matter, *W* sediment wetness, *LOI* organic matter content, *FSF* fine sediment fraction content, *T* temperature, *S* salinity, *Eh* oxidation–reduction potential

### **Composition and structure of the macrozoobenthos**

A total of 20 taxa representing Bivalvia (*Cerastoderma glaucum*, *Limecola balthica*, *Mya arenaria*), Crustacea (*Amphibalanus improvisus*, *Bathyporeia pilosa*, *Corophium* sp., *Gammarus* sp., *Idotea* sp., *Jaera* sp., *Rhithropanopeus harrisii*, *Lekanesphaera hookeri*), Gastropoda (*Peringia* sp., *Radix labiata*, *Theodoxus fluviatilis*), Polychaeta (*Hediste diversicolor*, *Marenzelleria* sp., *Streblospio shrubsolii*), Oligochaeta, Nemertea and insect larvae were identified in samples of macrozoobenthos collected in the Puck Lagoon (Table 2; Table SII). The number of macrozoobenthic taxa, as well as the species composition, depended on the location of the sampling station: 15 taxa have been identified in the area of Osłonino and 17 in the area of Chałupy. The occurrence of 12 macrozoobenthic taxa, *C. glaucum*, *M. arenaria*, *Corophium* sp., *Gammarus* sp., *Idotea* sp., *R. harrisii*, *Peringia* sp., *H. diversicolor*, *Marenzelleria* sp., Oligochaeta, Nemertea and insect larvae, was recorded at both research stations (Table SII).

**Table 2:** Feeding mode, trophic group and trophic level of macrozoobenthos species in the coastal zone of the Puck Lagoon (southern Baltic Sea) in years 2011–2013. The data on the *δ*^15^N used to calculate the tropic level can be found in Table SI and Fig. SI

	Food type	Feeding mode/habit	Trophic group	Tropic level
Bivalvia				
*Cerastoderma glaucum* (Bruguière, 1789)	POM, microalgae	Obligatory suspension feeder	Suspensivore	2.09
*Limecola balthica* (Linnaeus, 1758)	Microalgae, POM, SOM	Facultative suspension/deposit feeder	Suspensivore/detritivore	2.32
*Mya arenaria* (Linnaeus, 1758)	POM, microalgae	Obligatory suspension feeder	Suspensivore	2.35
Crustacea				
*Amphibalanus improvisus* (Darwin, 1854)	Microalgae, POM	Suspension feeder	Suspensivore	2.00
*Bathyporeia pilosa* (Lindström, 1855)	Microalgae, SOM	Facultative grazer/deposit feeder	Grazer	2.18
*Corophium* sp. (Latreille, 1806)	SOM, microalgae	Facultative deposit/suspension feeder/grazer	Suspensivore/detritivore	1.94
*Gammarus* sp. (Fabricius, 1775)	SOM, microalgae, live and dead animal tissue	Deposit feeder	Omnivore	2.12
*Idotea* sp. (Fabricius, 1798)	Microalgae, macroalgae	Herbivore	Grazer	2.32
*Jaera* sp. (Leach, 1814)	Microalgae, SOM	Herbivore/facultative deposit feeder	Grazer	1.78
*Rhithropanopeus harrisii* (Gould, 1841)	SOM, microalgae, live and dead animal tissue	Deposit feeder	Omnivore	2.32
*Lekanesphaera hookeri* (Leach, 1814)	Microalgae, macroalgae	Herbivore	Grazer	2.53
Gastropoda				
*Peringia* sp. (Hartmann, 1821)	Microalgae, SOM	Herbivore/facultative deposit feeder	Grazer	2.15
*Radix labiata* (Rossmässler, 1835)	Microalgae, macroalgae, SOM	Herbivore/facultative deposit feeder	Grazer	1.91
*Theodoxus fluviatilis* (Linnaeus, 1758)	Microalgae, macroalgae, SOM	Herbivore/facultative deposit feeder	Grazer	1.79
Polychaeta				
*Hediste diversicolor* (Müller, 1776)	SOM, microalgae, live and dead animal tissue	Facultative deposit feeder/predator/scavenger	Omnivore	2.56
*Marenzelleria* sp. (Mesnil, 1896)	Microalgae, SOM, live and dead animal tissue	Facultative deposit feeder/predator/scavenger	Omnivore	2.71
*Streblospio shrubsolii* (Buchanan, 1890)	SOM, microalgae	Facultative deposit/suspension feeder	Suspensivore/detritivore	nd
Oligochaeta	Bacteria, SOM	Deposit feeder	Detritivore	2.09
Nemertea	Live and dead animal tissue	Predator/scavenger	Omnivore	2.21
Insect larvae	Microalgae, live and dead animal tissue	Facultative deposit feeder/predator/scavenger	Omnivore	2.85

*nd* no data

In the area of the Osłonino station, eight taxa were classified as being euconstant or constant (frequency of occurrence higher than 50%): *L. balthica*, *Gammarus* sp., *Peringia* sp*.*, Oligochaeta, *Corophium* sp*.*, *H. diversicolor*, insect larvae and *M. arenaria* (Table SII)*.* The remaining seven species were counted as an accessory or accidental species. The dominant taxa in terms of abundance at the research area in Osłonino were *Peringia* sp*.* (38.0%) and *Corophium* sp*.* (31.0%). In terms of biomass, they were *H. diversicolor* (31.1%) and *Corophium* sp*.* (30.4%). At the station in Chałupy, 11 taxa of macrozoobenthos were euconstant or constant: *H. diversicolor*, *C. glaucum*, *Gammarus* sp*.*, *Peringia* sp*.*, Oligochaeta, *L. hookeri*, *Idotea* sp*.*, *T. fluviatilis*, insect larvae, *Corophium* sp. and Nemertea. The remaining six species were counted as an accessory or accidental species. In terms of numbers in the area of the Chałupy station, *Peringia* sp. (50.1%) and Oligochaeta (21.1%) were the dominant taxa. In macrozoobenthic biomass, however, *H. diversicolor* (33.1%) and *Peringia* sp. (17.5%) were dominant (Table SII).

### **Concentration of Hg in the diet components of the macrozoobenthos**

Hg_TOT_ level in the elements of macrozoobenthos diet collected in the Puck Lagoon varied in a wide range depending on the type of food, and the differences between concentrations measured in particular types were statistically significant (Kruskal–Wallis test, *p* = 0.00) (Table 3). Hg_TOT_ in the investigated components increased in the following order—at Osłonino station: sediments < vascular plants < macroalgae < epilithon < SPM < FLSM < epiphyton < phytoplankton < zooplankton (Kruskal–Wallis test, *p* = 0.00) and at Chałupy station: sediments < vascular plants < macroalgae < SPM < epilithon < phytoplankton < FLSM < epiphyton < zooplankton (Kruskal–Wallis test, *p* = 0.00).

**Table 3:** Mercury concentration in the various benthic components of the Puck Lagoon (southern Baltic Sea) in years 2011–2013

Station	Basic statistics	Suspended particulate matter		Surface sediments (the top 10 cm)			FLSM	Macro- algae	Vascular plants	Epilithon	Epiphyton	Phyto- plankton	Zoo-plankton	Fish
		Hg_TOT_ (ng g^−1^)	Hg_TOT_ (ng L^−1^)	Hg_TOT_ (ng g^−1^)	Hg_LOI_ (ng g^−1^)	Hg_FSF_ (ng g^−1^)	Hg_TOT_ (ng g^−1^)	Hg_TOT_ (ng g^−1^)	Hg_TOT_ (ng g^−1^)	Hg_TOT_ (ng g^−1^)	Hg_TOT_ (ng g^−1^)	Hg_TOT_ (ng g^−1^)	Hg_TOT_ (ng g^−1^)	Hg_TOT_ (ng g^−1^)
Osłonino	N	54	54	90	90	90	39	75	60	42	44	28	28	18
	Mean	47.3	2.3	2.8	334.4	330.9	48.8	16.7	8.2	31.0	64.9	115.0	132.3	71.5
	Median	44.8	1.2	2.6	332.5	186.9	48.1	17.5	8.0	26.2	66.9	66.8	78.3	69.5
	Min	5.6	0.2	0.6	75.0	16.4	26.1	7.4	2.8	10.5	49.7	1.0	16.2	49.4
	Max	109.8	10.0	7.9	935.0	965.0	64.0	24.8	12.9	69.0	78.3	631.4	596.8	89.6
Chałupy	N	51	51	90	90	90	42	84	96	36	44	30	30	27
	Mean	35.7	0.6	0.8	178.6	250.7	52.7	15.3	8.3	28.6	61.4	49.1	72.8	66.0
	Median	21.3	0.3	0.7	185.6	229.6	54.5	16.1	7.9	26.5	59.5	41.5	66.0	64.9
	Min	7.8	0.1	0.4	64.0	138.7	27.0	3.6	5.1	15.6	49.4	18.5	19.5	50.6
	Max	192.0	3.6	1.8	390.8	475.9	86.6	44.3	15.4	49.1	75.9	90.4	203.7	83.8

*N* number of analysed samples (including the number of repetitions: three in the case of suspended particulate matter and biological samples, five in the case of sediments), *FLSM* fluffy layer suspended matter, *Hg*_*TOT*_ total mercury, *Hg*_*LOI*_ and *Hg*_*FSF*_ mercury normalised to the content of LOI (organic matter content) and FSF (fine sediment fraction content) (Eqs. 3 and 4)

The lowest values were measured in the surface sediments in the case of both sampling stations. However, Hg_TOT_ concentration in sediments collected in Osłonino was significantly higher (median 2.6 ng g^−1^) than in Chałupy (median 0.7 ng g^−1^) (*U* Mann–Whitney test, *p* = 0.00) (Table 3). Relatively low levels of Hg_TOT_ were measured in macrophytobenthos. In the case of vascular plants, Hg_TOT_ concentrations in samples from Osłonino (median 8.0 ng g^−1^) were similar to the values in Chałupy (median 7.9 ng g^−1^) (*U* Mann–Whitney test, *p* = 0.79), as in the case of macroalgae, in which Hg_TOT_ concentrations measured in Osłonino (median 17.5 ng g^−1^) were only slightly higher than those in Chałupy (median 16.1 ng g^−1^) (*U* Mann–Whitney test, *p* = 0.14). In microphytobenthos, Hg_TOT_ concentrations were higher than those measured in macrophytes, especially in the case of epiphytes, in which measured values were over three times greater, both in Osłonino (median 66.9 ng g^−1^) and Chałupy (59.5 ng g^−1^). Similar to macrophytobenthos, Hg_TOT_ concentrations in benthic microflora did not differ statistically significant at the sampling stations both in epilithon (*U* Mann–Whitney test, *p* = 0.73) and epiphyton (*U* Mann–Whitney test, *p* = 0.23). Significant differences between Osłonino and Chałupy stations were noted in the case of an important food source, the suspended particulate matter (*U* Mann–Whitney test, *p* = 0.03). Hg_TOT_ concentrations in SPM at Osłonino (median 44.8 ng g^−1^) were two times higher than values in Chałupy (median 21.3 ng g^−1^). Higher Hg_TOT_ levels in Osłonino (median 66.8 ng g^−1^) in comparison to Chałupy (median 41.5 ng g^−1^) were also measured in other significant component of macrofauna diet, which is phytoplankton (*U* Mann–Whitney test, *p* = 0.04). The highest Hg_TOT_ level was noted in zooplankton, both in the case of the station in Osłonino (median 78.3 ng g^−1^) and in Chałupy (median 64.9 ng g^−1^) (Table 3).

### **Concentration of Hg in the macrozoobenthos**

Hg_TOT_ concentrations in the macrozoobenthos of the Puck Lagoon ranged from 7.4 ng g^−1^ to 521.2 ng g^−1^, wherein the values measured in individual species differed statistically (Kruskal–Wallis test, *p* = 0.00) (Table 4). The highest median concentration of Hg_TOT_ was measured in *B. pilosa* (471.3 ng g^−1^) collected in Osłonino, while the lowest were noted in *A. improvisus* (4.1 ng g^−1^) collected in Chałupy. However, both taxa were classified as accidental, which means they had low frequency of occurrence in the studied area (Table SII).

**Table 4:** Mercury concentration in particular species of macrofauna of the Puck Lagoon (southern Baltic Sea) in years 2011–2013

Station	Basic statistics	Bivalvia			Crustacea								Gastropoda			Polychaeta			Oligochaeta	Nemertea	Insect larvae
		*Cerastoderma glaucum*	*Limecola baltica*	*Mya arenaria*	*Amphibalanus improvisus*	*Bathyporeia pilosa*	*Corophium sp*.	*Gammarus sp*.	*Idotea sp*.	*Jaera sp*.	*Rhithropanopeus harrisii*	*Lekanesphaera hookeri*	*Peringia sp*.	*Radix labiata*	*Theodoxus fluviatilis*	*Hediste diversicolor*	*Marenzelleria sp*	*Streblospio shrubsolii*			
Osłonino	N	18	24	13	nd	1	27	28	9	nd	1	nd	36	nd	nd	33	11	1	12	1	12
	Mean	72.8	51.0	12.8		471.3	41.0	59.3	86.9		71.9		56.2			37.8	49.1	24.3	50.9	38.7	49.7
	Median	74.7	49.2	14.0		471.3	42.2	28.6	26.7		71.9		54.1			28.9	48.9	24.3	36.3	38.7	50.3
	Min	18.4	26.8	7.4			25.4	8.2	22.0				26.2			9.3	19.5		8.7		10.9
	Max	123.3	74.4	20.6			60.8	334.6	410.2				110.5			157.4	83.4		223.4		97.9
Chałupy	*N*	22	nd	12	1	nd	16	26	17	1	1	12	26	4	10	14	1	nd	13	8	13
	Mean	32.8		62.8	4.1		65.3	44.6	38.3	151.8	57.2	55.9	77.6	63.4	63.8	22.8	415.4		72.3	189.3	61.4
	Median	22.5		65.7	4.1		34.9	31.6	37.1	151.8	57.2	26.6	59.8	64.4	49.9	21.6	415.4		21.8	59.2	17.9
	Min	12.7		54.4			8.5	10.9	14.9			15.7	28.9	32.3	30.7	12.6			8.6	23.2	14.9
	Max	71.6		76.9			213.7	170.1	65.6			177.6	185.6	92.8	140.5	39.7			521.2	460.2	177.9

*N* number of analysed samples (including the number of repetitions: from 1 to 5, depending on the weight of organisms from a given species), *nd* no data (the species was not observed or the mass of the sample was insufficient for analysis)

**Fig. 2:**
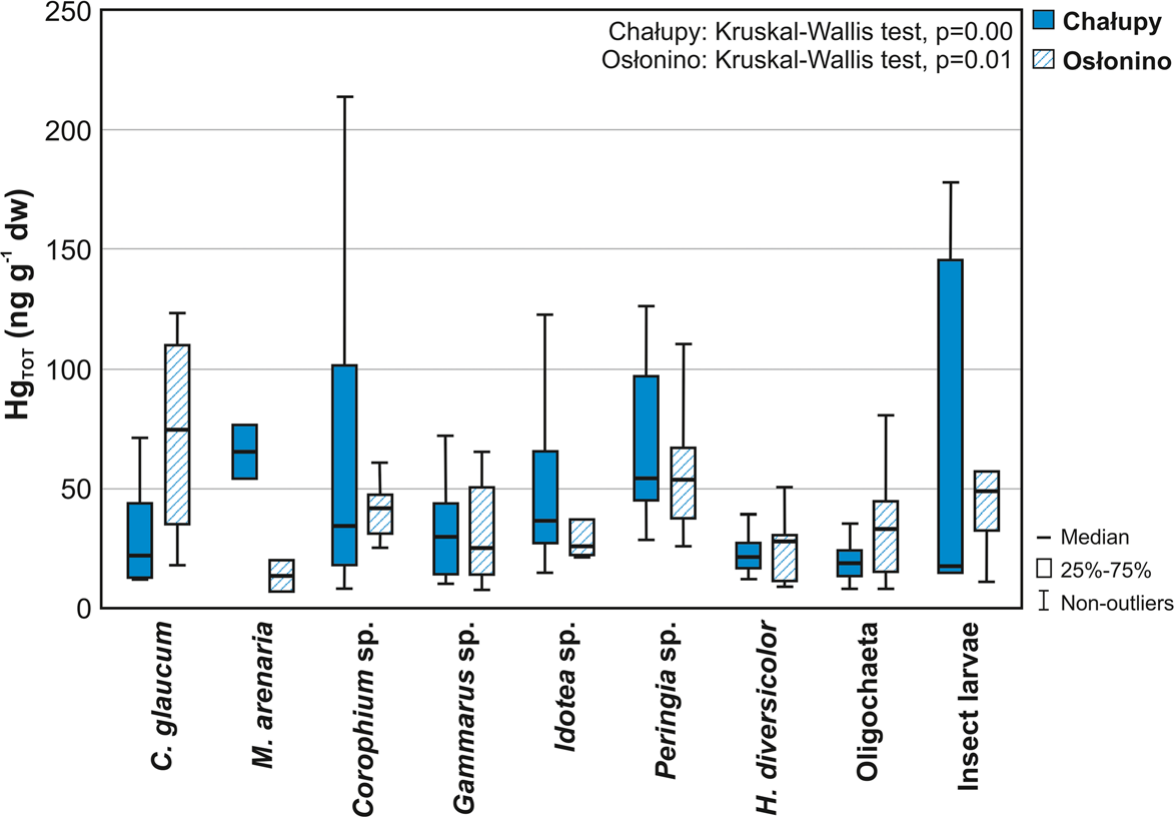
Total mercury (Hg_TOT_) concentration in macrozoobenthos of the coastal zone of the Puck Lagoon (southern Baltic Sea) in years 2011–2013 (species common to both stations, excluding accidental taxa)

Considering only the Hg_TOT_ level in nine constant or euconstant species (after rejection of accessory and accidental taxa) common to both research stations (*C. glaucum*, *M. arenaria*, *Corophium* sp*.*, *Gammarus* sp*.*, *Idotea* sp*.*, *Peringia* sp*.*, *H. diversicolor*, Oligochaeta and insect larvae), the differences between concentrations measured in Chałupy and Osłonino were not statistically significant (*U* Mann–Whitney test, *p* = 0.50). However, the distribution of Hg_TOT_ concentrations in individual species at these stations was diverse (Fig. 2). Hg_TOT_ in the investigated organisms increased in the following order—at Osłonino station: *M. arenaria* < *Idotea* sp*.* < *Gammarus* sp*.* < *H. diversicolor* < Oligochaeta < *Corophium* sp*.* < insect larvae **<** *Peringia* sp*.* < *C. glaucum* (Kruskal–Wallis test, *p* = 0.01) and at Chałupy station: insect larvae **<** *H. diversicolor* < Oligochaeta < *C. glaucum* < *Gammarus* sp*.* < *Corophium* sp*.* < *Idotea* sp*.* < *Peringia* sp*.* < *M. arenaria* (Kruskal–Wallis test, *p* = 0.00) (Fig. 2; Table 4). On the basis of inter-species variation of Hg_TOT_ level (multiple comparison of ranks), the three groups of organisms were separated. In the area of Osłonino, the first group with the highest concentrations of Hg_TOT_ (consisted of two taxa: *C. glaucum* (median 74.7 ng g^−1^) and *Peringia* sp. (median 54.3 ng g^−1^), the second group with moderate Hg_TOT_ level was represented by three taxa: insect larvae (median 50.3 ng g^−1^), *Corophium* sp. (median 42.2 ng g^−1^) and *Idotea* sp. (median 26.7 ng g^−1^), while the lowest Hg_TOT_ concentrations were measured in third group consisting of four taxa: Oligochaeta (median 36.3 ng g^−1^), *Gammarus* sp. (median 28.6 ng g^−1^), *H. diversicolor* (median 28.9 ng g^−1^) and *M. arenaria* (median 14.0 ng g^−1^). The significant differences in the Hg_TOT_ concentrations were observed between first and third group (Dunn’s test, *p* = 0.00). In the case of the Chałupy station, the first group of constant taxa with the highest Hg_TOT_ was *M. arenaria* (median 65.7 ng g^−1^) and *Peringia* sp. (median 59.8 ng g^−1^), the second group was consisting of five taxa: *Idotea* sp. (median 37.1 ng g^−1^), *Corophium* sp. (median 34.9 ng g^−1^), *Gammarus* sp. (median 31.6 ng g^−1^), *C. glaucum* (median 22.5 ng g^−1^) and insect larvae (median 17.9 ng g^−1^), while the third group was represented by Oligochaeta (median 21.8 ng g^−1^) and *H. diversicolor* (median 21.6 ng g^−1^). The significant differences in the Hg_TOT_ concentrations were observed between every pair of groups: between first and second (Dunn’s test, *p* = 0.02), first and third (*p* = 0.00) and second and third (*p* = 0.04).

**Fig. 3:**
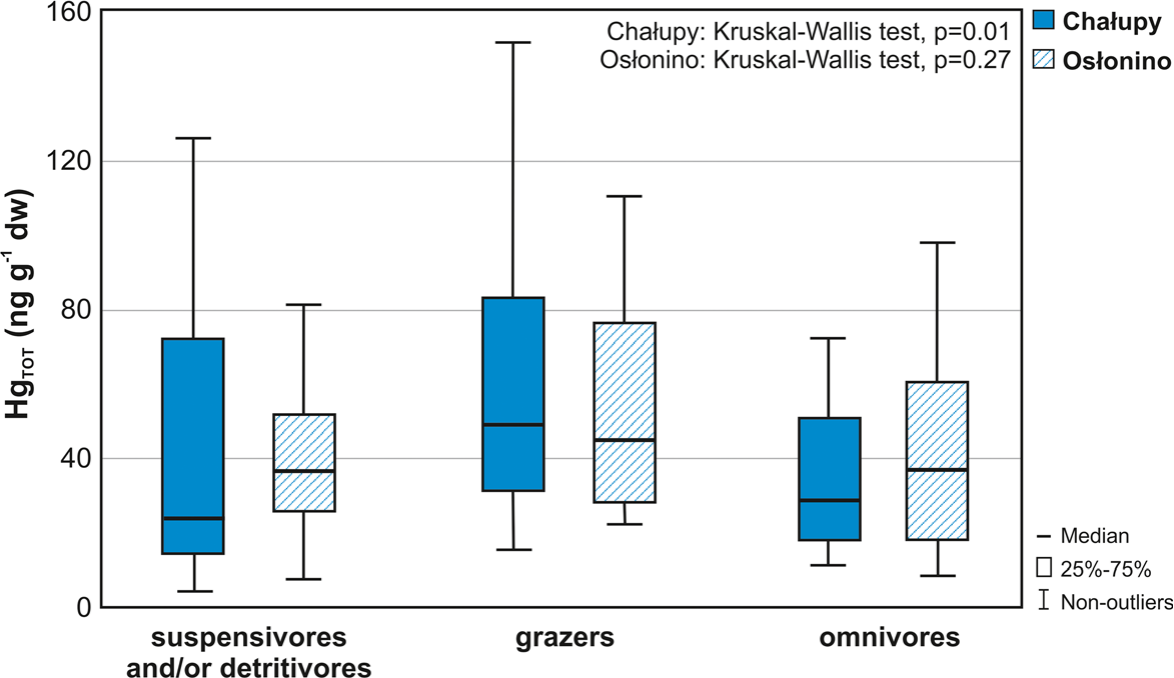
Total mercury (Hg_TOT_) concentration in the main trophic groups of macrozoobenthos of the coastal zone of the Puck Lagoon (southern Baltic Sea) in years 2011–2013

Taking into account the feeding mode of the macrofauna (Table 2), the collected organisms were assigned to three trophic groups: suspensivores and/or detritivores, grazers and omnivores. Concentration of Hg_TOT_ in these groups increased in the following order—at Osłonino station: omnivores (37.7 ng g^−1^) < suspensivores and/or detritivores (38.7 ng g^−1^) < grazers (50.0 ng g^−1^) (Kruskal–Wallis test, *p* = 0.27) and at Chałupy suspensivores and/or detritivores (24.3 ng g^−1^) < omnivores (28.9 ng g^−1^) < grazers (51.2 ng g^−1^) (Kruskal–Wallis test, *p* = 0.01) (Fig. 3). The significant differences in the Hg_TOT_ concentrations measured in the different trophic groups of macrozoobenthos were noted only at Chałupy station: between suspensivores and/or detritivores and grazers (Dunn’s test, *p* = 0.02) and between grazers and omnivores (*p* = 0.00).

## Discussion

### **Spatial variability of Hg concentrations**

The varied structure of zoobenthos at research stations in the Puck Lagoon was primarily related to the nature and configuration of the bottom of each station, as well as to the influence of local runoff (Kotwicki 1997; Janas and Kendzierska 2014). The research stations at Osłonino and Chałupy, despite being located only a short distance from each other (10 km in a straight line) (Fig. 1), differed in terms of prevailing environmental conditions and the parameters of surface sediments (Table 1). These differences were largely dependent on the location of the station and hence the distance from local sources of organic and inorganic matter (Jędruch et al. 2017).

#### The inner part of a semi-enclosed gulf—Osłonino

The Osłonino station was located in the direct impact zone of land-based sources. The increased surface runoff was related to the proximity of river estuaries and coastal abrasion (Fig. 1), and the limited water dynamics were conducive to the accumulation of organic matter (Bełdowska et al. 2016a; Jędruch et al. 2017; Kwasigroch et al. 2018). This resulted in the high concentration of SPM in the area of Osłonino (Table 1) and its isotopic composition, indicating terrigenous origin (median *δ*^13^C − 24.3‰; median *δ*^15^N 4.2‰; median C_ORG_/N_TOT_ 9.7) (Jędruch et al. 2017) (Table SI). Low water dynamics in the area of the Osłonino station allowed sediments of a smaller size to be deposited there more than at the Chałupy station (Table 1). The availability of food in the form of particulate organic matter (POM) and sediment organic matter (SOM) has contributed to the development of suspension/deposit feeders such as *Corophium* sp. (crustacean), *C. glaucum* and *L. balthica* (clams) and Oligochaeta (Table 2), whose share in the zoobenthos biomass in the area of Osłonino was greater than in Chałupy (Table SII).

Increased surface runoff and accumulation of matter in the coastal zone in the vicinity of the station also influenced higher Hg_TOT_ concentrations in SPM and surface sediments, as well as in the analysed biotic components, phytobenthos, epilithon, epiphyton and plankton (Table 3), compared to the Chałupy station situated away from land sources. In nine macrozoobenthic taxa, common to both research stations (Table SII), higher Hg_TOT_ concentrations were observed in the area of Osłonino than Chałupy in five taxa: *C. glaucum*, insect larvae, Oligochaeta, *H. diversicolor* and *Corophium* sp. (Fig. 2). The biggest difference was observed in the suspension feeder *C. glaucum*, in which the concentration of Hg_TOT_ in Osłonino (median 74.7 ng g^−1^) was more than three times higher than in Chałupy (median 22.5 ng g^−1^) (Fig. 2; Table 4). The diet of this species consists of microalgae (including phytoplankton, microphytobenthos, and epiphytes) and SPM (Rossi et al. 2004; Olenin and Daunys 2005). High concentrations of Hg_TOT_ in *C. glaucum* in Osłonino were connected with elevated metal concentrations in its food sources. This is confirmed by correlation of Hg_TOT_ concentration in *C. glaucum* and in phytoplankton (*R* = 0.67) and correlation of Hg_TOT_ level in *C. glaucum* and SPM (*R* = 0.43) (Table SIII). In the case of phytoplankton, the Hg_TOT_ concentration at the Osłonino station was about 60% higher than at the Chałupy station, and the concentration of Hg_TOT_ in SPM in Osłonino was three times higher than in Chałupy (Table 3). A significant difference was also observed in the case of omnivorous insect larvae, for which the concentration of Hg_TOT_ in Osłonino (median 49.6 ng g^−1^) was about 2.5 times higher than in Chałupy (median 17.9 ng g^−1^) (Table 4). Due to the fact that insect larvae have a very diverse diet, including microalgae, live and dead animal tissue and sometimes also detritus (Table 2) (Sanseverino and Nessimian 2001; Pekcan-Hekim et al. 2006), no statistically significant correlation was observed between the Hg_TOT_ concentration in these organisms and the Hg_TOT_ concentration in any of their dietary components. Nevertheless, the elevated Hg_TOT_ concentration in insect larvae in Osłonino can be attributed to higher Hg_TOT_ concentrations in their potential sources of food there than at Chałupy (Table 3). Other taxa for which the concentration of Hg_TOT_ was higher in the area of Osłonino were Oligochaetes, *Corophium* sp. and *H. diversicolor*; however, the noted differences did not exceed 65% of the concentrations measured in these species in the Chałupy region (Table 4)*.* As in the case of *C. glaucum* and insect larvae, the higher Hg_TOT_ levels, measured in Oligochaetes, *Corophium* sp. and *H. diversicolor* in Osłonino, were associated with increased Hg_TOT_ concentration in their diet (Table 3; Table SIII)—the surface sediments in the case of Oligochaetes (van de Bund et al. 1994; Rodrigues and Reynoldson 2011); suspended matter, microalgae and sediments in the case of *Corophium* sp. (Olenin and Daunys 2005; Riisgård and Schotge 2007); and sediments and microalgae in the case of *H. diversicolor* (Olivier et al. 1995; Olenin and Daunys 2005).

#### Part of the gulf under little land influence—Chałupy

In the area of the Chałupy station, the inflow of material from land-based sources was limited. This is confirmed by the lower concentration of SPM and the lower share of organic matter and fine particle fraction in surface sediments, in comparison with Osłonino (Table 1). What is important, the Chałupy site was not only characterised by a different amount of matter compared to the Osłonino site but, more importantly, typically marine origin (median *δ*^13^C − 22.0‰; median *δ*^15^N 3.1‰; median C_ORG_/N_TOT_ 8.1) (Jędruch et al. 2017) (Table SI). This material comes from in situ primary production (mainly microalgae and phytoplankton) but is also transported from the deeper parts of the Gulf of Gdańsk (Jędruch et al. 2017). The station in Chałupy was also rich in bottom vegetation—macroalgae and vascular plants. This ensured that the biomass of phytobenthos in the Chałupy region was almost double that of the station in Osłonino (Bełdowska et al. 2016b). This resulted in richer species composition and increased biomass of grazing zoobenthic organisms, such as molluscs: *Peringia* sp., *T. fluviatilis* and *R. labiata* and crustaceans: *Idotea* sp. and *L. hookeri*, in comparison with the station at Osłonino (Table 2; Table SII).

Among the taxa noted at both stations, higher Hg_TOT_ concentrations at the Chałupy station in comparison to the Osłonino station were observed in four taxa: *M. arenaria*, *Idotea* sp., *Peringia* sp. and *Gammarus* sp. (Fig. 2; Table 4). The greatest difference was observed in the suspensivore *M. arenaria*, for which the Hg_TOT_ concentrations at the Chałupy station (median 65.7 ng g^−1^) were more than four times higher than at the station in Osłonino (median 14.0 ng g^−1^). Although *M. arenaria* is a suspension feeder which feeds on suspended matter and microalgae, similar to *C. glaucum*, it also feeds on detritus and organic matter deposited in sediments (Bacon et al. 1998; Olenin and Daunys 2005) (Table 2). Hg_TOT_ concentrations in this species increased together with the share of fine fraction in surface sediments (*R* = 0.81) and Hg_TOT_ concentration in FLSM (*R* = 0.62) (Table SIII). Elevated Hg_TOT_ concentrations in *M. arenaria* may be due to the fact that the dominant form of Hg in sediments in the Chałupy region was Hg adsorbed on the fine fraction of sediment (Hg_FSF_) (Table 4). This confirms the relationship between the concentration of Hg_TOT_ in surface sediments and contents of the FSF (*R* = 0.80). This may indicate the removal of Hg-rich fine fraction sediments deposited in the deeper parts of the Gulf of Gdańsk (e.g. as a consequence of upwelling) and their transport to the shallow Puck Lagoon (Jędruch et al. 2015; Jędruch et al. 2017). The other taxa which were characterised by slightly higher (about 10%) Hg_TOT_ concentrations at the Chałupy station were *Idotea* sp., *Peringia* sp., and *Gammarus* sp. (Fig. 2; Table 4). In the case of grazing *Idotea* sp. and *Peringia* sp. (Kamermans et al. 2002; Riera 2010), the elevated Hg_TOT_ concentration was correlated with the Hg_TOT_ concentration in benthic macrophytes and suspended matter (Table 3; Table SIII). In the case of omnivorous *Gammarus* sp., no relationship was observed between the Hg_TOT_ in these taxa and in its potential food sources.

### **Hg bioaccumulation and biomagnification**

The Hg_TOT_ concentrations in macrofauna varied according to the diet of the analysed species. The highest Hg_TOT_ levels, at both research stations, were measured in grazers (Fig. 3), which feed mostly on the microscopic algae, diatoms and detritus which createa biofilm that covers the macrophytes, rocks and other underwater surfaces (Table 2). High Hg_TOT_ concentrations in grazing macrofauna compared to other trophic groups were most distinct at the Chałupy station—the concentration of Hg_TOT_ in grazers was about 2-fold higher than in those feeding on suspension and/or detritus and omnivores. In the case of the station in Osłonino, the concentration of Hg_TOT_ in grazers was similar to that measured in Chałupy (*U* Mann–Whitney test, *p* = 0.76); however, the differences between grazers and other trophic groups in Osłonino were slight (Fig. 3). Such a distribution of Hg_TOT_ concentrations in the macrozoobenthic trophic groups is related to the concentration of Hg_TOT_ in the food components of the bottom fauna. This was confirmed by the concentrations of Hg_TOT_ in benthic micro-organisms forming the basic diet of grazers (epiphyton), which were higher than, for example, those measured in SPM—the main dietary component for filter organisms (Table 3). Additionally, as shown by studies conducted in the Puck Lagoon by Jankowska et al. (2018), an important source of food for grazers, along with microphytobenthos, may also be sedimentary organic matter (FLSM), in which concentrations were close to those measured in epilithic algae (Table SII). It may also be related to the form of Hg in the examined food sources—about 90% of Hg accumulated in benthic flora of the Puck Lagoon is in bioavailable labile form, while in the case of surface sediments only a half of Hg can be introduced to the trophic chain (Jędruch, unpublished).

#### **BSAF**

**Fig. 4:**
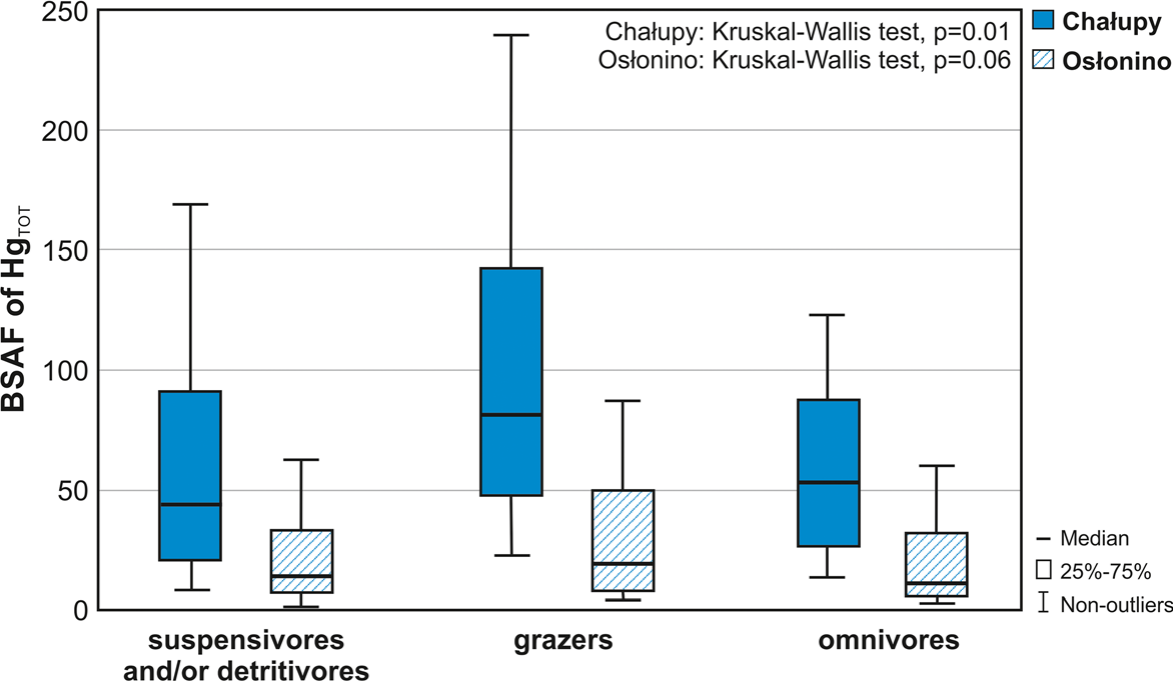
Biota–sediment accumulation factor (BSAF) of total mercury (Hg_TOT_) in the main trophic groups of macrozoobenthos of the coastal zone of the Puck Lagoon (southern Baltic Sea) in years 2011–2013

An additional indicator for a better understanding of the complex mechanism for incorporating Hg_TOT_ into the marine trophic chain is the BSAF, a single-compartment model that predicts partitioning between exposure medium (sediments) and biota (McGeer et al. 2003). This parameter differed statistically in the case of research stations in the Puck Lagoon (*U* Mann–Whitney test, *p* = 0.00), with the values calculated for Chałupy being about four times higher (median 61.3) than in Osłonino (median 15.1) (Fig. 4). This was primarily related to the environmental conditions prevailing at the research stations. Numerous worldwide studies, in both marine and freshwater environments, indicate a significant effect of aerobic conditions on the uptake of Hg by organisms on the lowest trophic level (Sokołowski 2009; Wyn et al. 2009; Kidd et al. 2012). Lower oxygenation of surface sediments in the area of Osłonino is confirmed by lower values of potential Eh in comparison with the values measured in Chałupy (Table 1). Moreover, in the area of Osłonino, a complete depletion of oxygen and the temporary appearance of H_2_S was noted periodically (Bełdowska et al. 2013; Jędruch et al. 2018a). As the oxidation of sediments decreases, the abundance of anaerobic bacteria increases, and this is accompanied by a drop in Hg concentration in pore waters (Bełdowska et al. 2013). As a consequence, the accumulation of Hg in zoobenthic organisms is limited. In addition to this, the limited accumulation of Hg in the macrozoobenthos at Osłonino could be associated with lower pH and higher water temperature in comparison to Chałupy (Table 1), especially in the summer season (mean in the summer season 20.0 °C and 17.8 °C, respectively). This was also indicated by the research results of Greenfield et al. (2001) and Lavoie et al. (2013). The higher temperature in Osłonino during the summer stimulated the growth of organisms (greater biomass of organisms at Osłonino than Chałupy), leading to the “dilution” of Hg accumulated in body tissues.

The limited accumulation of Hg in macrozoobenthos in Osłonino could also be connected to an increased inflow and accumulation of organic matter in the area of the station, as well as increased nutrient supply associated with increased surface runoff (Kidd et al. 2012; McGeer et al. 2003; Jędruch et al. 2017). This is confirmed by the results of experimental research conducted by Pickhardt et al. (2002), concerning the effect of the increased load of biogenic substances and subsequent algal blooms. More specifically, while algae effectively and rapidly concentrate both inorganic and organic Hg, the metal burden per cell decreases in algal blooms. This results in diluted concentrations of bioavailable Hg in algae, thus limiting its transfer to the organisms which feed on it. Similar results were noted by Chouvelon et al. (2018) in the comparative study of two marine ecosystems, oligo- and mesotrophic ones. In the case of area with higher productivity, the bioaccumulation of Hg in medium-trophic level consumers was lower compared to the organisms from oligotrophic waters.

The comparison of BSAF values at two research stations showed the significant impact of the feeding mode and activity of macrofauna on the accumulation of Hg. The bioaccumulation of Hg_TOT_, both at Chałupy and Osłonino, was found to be the greatest in the omnivorous group (Fig. 4), a group dominated by taxa burrowing deeply in sediments, such as *H. diversicolor* or *Marenzelleria* sp. (Leppakoski and Olenin 2000). Higher BSAF in burrowing macrofauna compared to epifauna living on the sediment’s surface can be associated with the release of Hg accumulated in the deeper layers of sediments. Although the Hg in the deeper sediments occurs mostly as a stable HgS, as a result of bioturbation and increased oxygen input, it may transform to bioavailable Hg sulphate (Bełdowski and Pempkowiak 2003).

#### **BMF**

Hg_TOT_ concentrations measured in individual elements of the coastal zone increased together with trophic level. The linear function of ln-transformed Hg_TOT_ concentration in individual environmental elements and values of *δ*^15^N representing the trophic position (*R* = 0.51, *p* < 0.001) (according to Eq. 9) points to Hg biomagnification in the trophic chain, as testified to by the positive slope coefficient value for the function (Lavoie et al. 2013; Riyadi et al. 2015). This coefficient, also known as the trophic magnification slope (TMS) (Nfon et al. 2008; Lavoie et al. 2013), calculated for data from the Puck Lagoon, was 0.22 (Table 5). This value was within the range of results obtained by other researchers of aquatic food webs (0.01–0.40) (i.e. Chen et al. 2000; Campbell et al. 2003; Al-Reasi et al. 2007; Lavoie et al. 2010; Chouvelon et al. 2018). The TMS in Puck Lagoon was higher than the mean (0.16) based on a compilation of data from more than 200 aquatic environments worldwide. However, the TMS in the Puck Lagoon was close to the mean calculated only for marine systems (0.20) (Lavoie et al. 2013) and particular marine systems located in the temperate zone (0.22) (Table 5; Fig. 6). The mean Hg biomagnification factor (BMF) calculated on the basis of the TMS value (Eq. 10) in the trophic web of the Puck Lagoon was 2.2 and was about 20% higher than the global average (1.8). This is connected to the relatively low productivity of the Puck Lagoon system, as well as to conditions related to the latitude—the higher values of BMF were calculated in temperate and polar regions in comparison to the tropic zone (Sokołowski et al. 2012; Lavoie et al. 2013; Riyadi et al. 2015).

**Table 5:** Comparison of trophic magnification slope (TMS) of total mercury (Hg_TOT_) in the marine systems around the world

Zone	Region	Country	TMS	Reference
Tropical	Gulf of Oman	Oman	0.07	Al-Reasi et al. 2007
	Sepetiba Bay	Brazil	0.07	Bisi et al. 2012
	Jakarta Bay	Indonesia	0.07	Riyadi et al. 2015
	Guanabara Bay	Brazil	0.18	Bisi et al. 2012
Temperate	Azores	Portugal	0.01	Newman et al. 2011
	Gulf of Lion	France	0.11	Chouvelon et al. 2018
	Masan Bay	Korea	0.12	Kim et al. 2012
	Northern Yellow Sea	China	0.13	Zhao et al. 2013
	Gulf of Mexico	USA	0.17	Cai et al. 2007
	Gulf of St Lawrence	Canada	0.17	Lavoie et al. 2010
	Puck Lagoon	Poland	0.22	This study
	Bird Island	South Georgia	0.27	Anderson et al. 2009
	Australian waters	Australia	0.31	Pethybridge et al. 2012
	Sanriku coast	Japan	0.33	Riyadi et al. 2015
	Gulf of the Farallones	USA	0.33	Jarman et al. 1996
	Bay of Biscay	France	0.35	Chouvelon et al. 2018
Polar	West Greenland	Greenland	0.08	Rigét et al. 2007
	Nasaruvaalik	Greenland	0.10	Clayden et al. 2015
	Alaskan Arctic	USA	0.10	Fox et al. 2017
	Lancaster	Canada	0.14	Atwell et al. 1998
	Northwater Polynya	Canada	0.20	Campbell et al. 2005
	Kongsfjorder, Svalbard	Norway	0.21	Jæger et al. 2009
	Amundsen Shelf	Canada	0.25	Loseto et al. 2008
	Icelandic waters	Iceland	0.26	McMeans et al. 2010
	Chukchi Sea	USA	0.29	Dehn et al. 2006

**Fig. 5:**
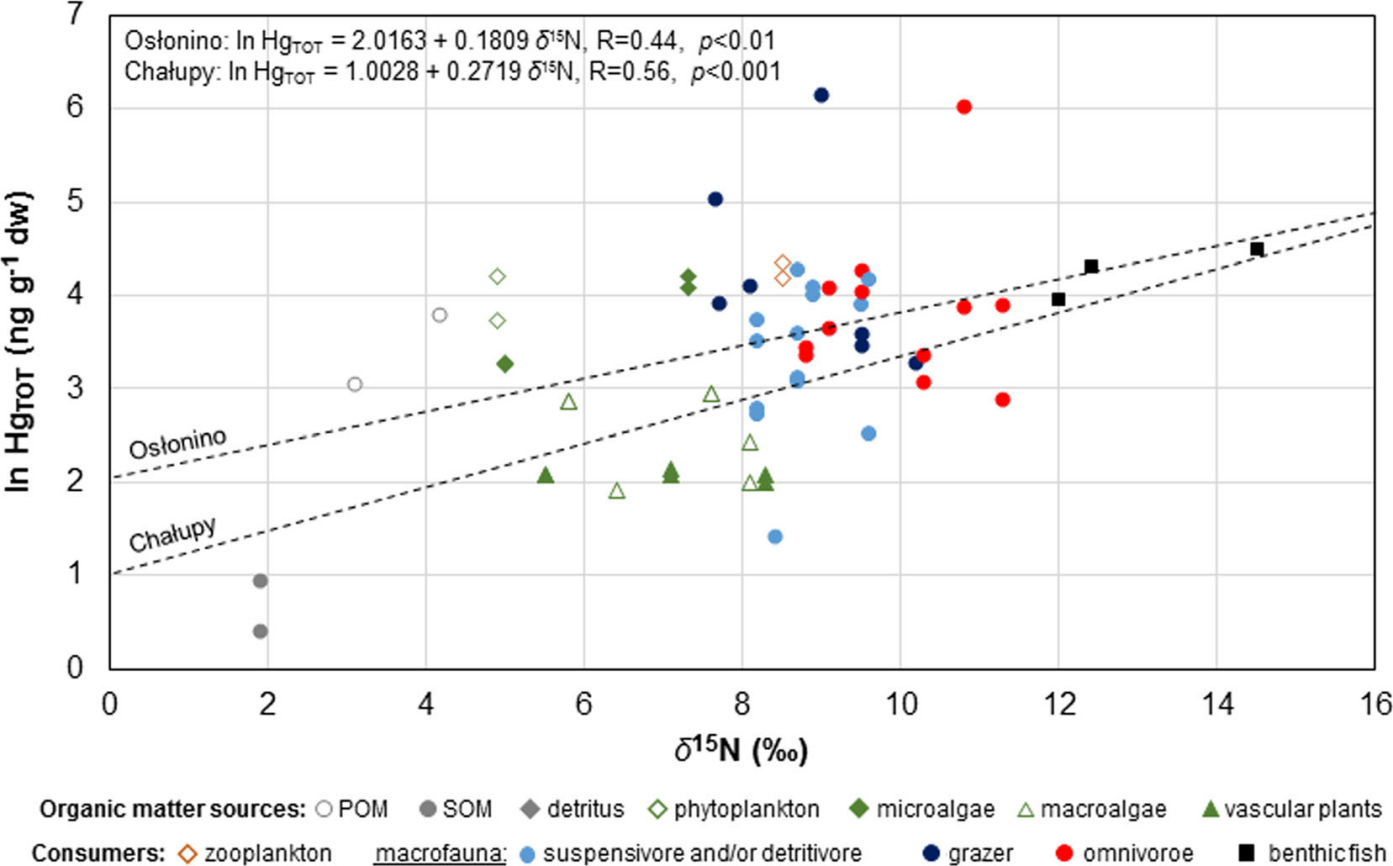
Trophic magnification slope (TMS) of total mercury (Hg_TOT_) calculated on the basis of the relationship between median values of ln-transformed Hg_TOT_ concentrations and *δ*^15^N in various components of the benthic food web from the sampling stations (Osłonino and Chałupy) in the coastal zone of the Puck Lagoon in years 2011–2013. The data on the *δ*^15^N can be found in Table SI and Fig. SI

**Fig. 6:**
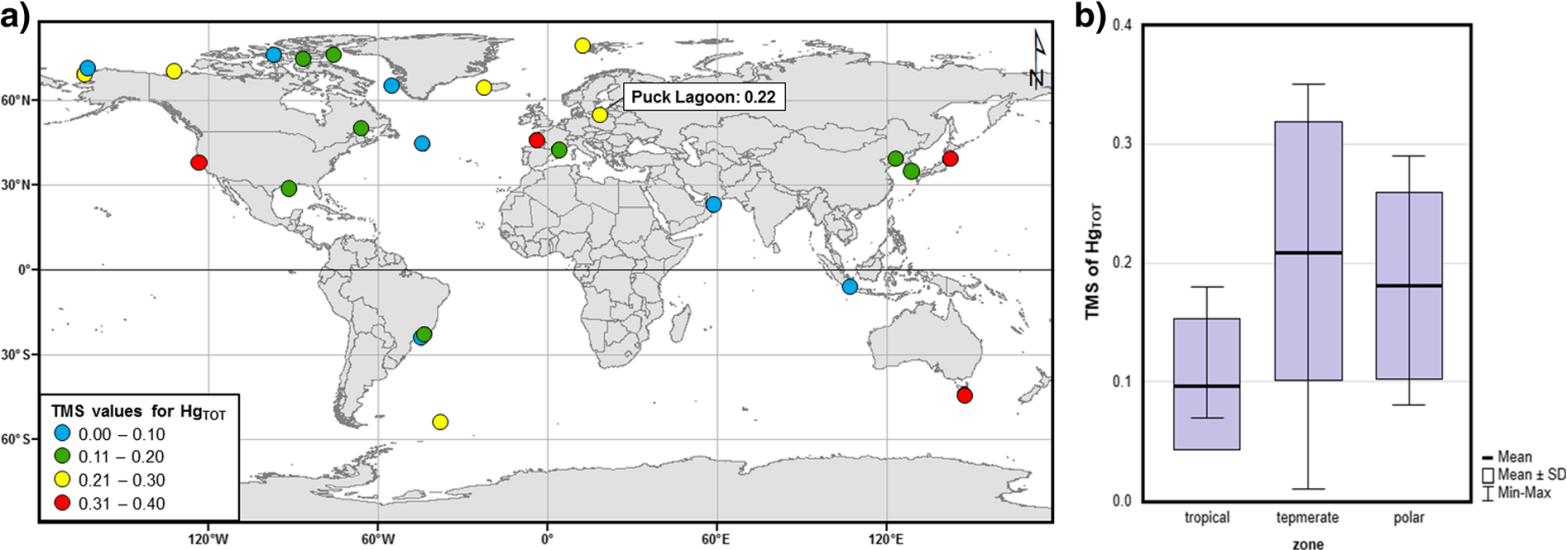
Trophic magnification slope (TMS) of total mercury (Hg_TOT_) in the marine systems around the world (**a**) together with basic statistics of TMS depending on the latitude (**b**) based on literature data (data sources can be found in Table 5)

Geographical differences in the Hg transfer rates were also reported for the research stations in the inner coastal zone of the Puck Lagoon. The TMS of Hg, calculated on the basis of ln-transformed Hg concentrations and *δ*^15^N values, in Osłonino was lower (0.18) in comparison to Chałupy (0.27) (Fig. 5), which indicated a different biomagnification power of Hg in the trophic chains at these station. The biomagnification factor of Hg calculated on the basis of TMS values was 1.8 in Osłonino and 2.5 in Chałupy (Fig. 6). These differences are related not only to the structure of the trophic web and to the dietary habits of primary consumers (Chouvelon et al. 2018) but also to the different environmental conditions prevailing at research stations in the coastal zone (Wyn et al. 2009; Kidd et al. 2012). Although the concentration of Hg in benthic organisms at the station in Osłonino was slightly higher than the station in Chałupy (Fig. 2; Tables 3 and 4), the rate of Hg biomagnification from food sources to consumers in Osłonino was slower. This could also be related to greater productivity in the Osłonino region, resulting in a higher biomass of producers and macrozoobenthic organisms, and also suspended organic matter (Bełdowska and Kobos 2016; Jędruch et al. 2017). Similar impact of the productivity of marine systems on Hg biomagnification was observed in the Mediterranean and Atlantic waters (Chouvelon et al. 2018). The trophic factor of waters strongly influences the Hg biomagnification, through the “biodilution effect” (Pickhardt et al. 2002). It is due to the higher number and higher surface area/volume ratio (i.e. size) of cells at the base of systems with high productivity. This configuration is less favourable to an efficient uptake of Hg by cells, whose the lower Hg burden is then transferred to consumers. Moreover, in areas with low primary production, where cells are thus less abundant and potentially contain higher Hg burden, primary consumers probably consume virtually all of them (Chouvelon et al. 2018). Biodilution of Hg in the macrofauna also occurs as a result of to the high abundance of detrital and organic particles and growth dilution (fast growth rates for longer lived organisms), across several trophic levels (Kidd et al. 2012). In addition to this, in waters of a lower pH, the lower Hg biomagnification may be due to the effect of water temperature or chemistry on the methylation of inorganic Hg to methylmercury and/or its availability to primary producers and consumers (Wyn et al. 2009; Chouvelon et al. 2018). Numerous studies have demonstrated the relationship between limited Hg biomagnification in the initial links of the trophic chain and low oxygen conditions, which are likely to occur at organic-rich regions of the inner Puck Lagoon, such as Osłonino. The periodic deficiency of oxygen and temporal presence of hydrogen sulphide at this station likely influenced Hg transfer in benthic communities by altering Hg bioavailability (Sokołowski 2009).

### **Temporal trends**

Species composition analysis demonstrated the restructuring of macrozoobenthos in the Puck Lagoon coastal zone, in comparison with previous years (e.g. Legeżyńska and Wiktor 1981; Kotwicki 1997; Janas and Kendzierska 2014). The observed changes may entail far-reaching alterations in the nature and rate of flow of chemicals (and hence Hg) through the various trophic levels and therefore the whole ecosystem.

In the case of the station in Osłonino, a particularly important observation is the increased percentage over the past decade or so of Crustacea in the macrozoobenthos biomass. In 2011–2013, this percentage share was about 2.5 times higher than in the early 1990s (Kotwicki 1997) (Table SII), and the concentration of Hg_TOT_ in this subphylum (mean 74.8 ng g^−1^) was found to be almost 30% higher than the metal level measured in all other macrofauna species in the area of this station (mean 59.6 ng g^−1^). Continuation of this upward trend in the percentage of Crustacea in the macrozoobenthos biomass, together with a decreasing share of groups with lower Hg_TOT_ concentrations (e.g. Bivalvia, Polychaeta) (Table 4), may increase a load of metal entering the trophic chain. Crustaceans such as *Gammarus* sp., *Idotea* sp. and *Corophium* sp. are important dietary constituents of the majority of Puck Lagoon fish, their offspring and higher-trophic-level crustaceans such as the *Crangon crangon* or *Palaemon* sp. varieties of shrimp (Morawski 1978; Legeżyńska and Wiktor 1981).

In the coastal zone at Chałupy, changes to the structure of zoobenthos became apparent especially in the case of Bivalvia. In the area of that station, the average share of this class of organisms in the biomass of bottom macrofauna in the years 2011–2013 was over two times lower than in 1992–1993 (Kotwicki 1997). Bivalvia once represented roughly half of the benthic macrofauna biomass, but now, their average share does not exceed 20% (Table SII). The drop in biomass of molluscs, with their high filtration capacity, can lead to changes in the flow rate of matter in the ecosystem, including an increased rate of accumulation of organic matter (and thus Hg) in bottom sediments (Błędzki and Kruk-Dowgiałło 1983). The concentration of Hg_TOT_ measured in Bivalvia (mean 42.2 ng g^−1^) was about 40% lower than the average metal concentration in macrozoobenthos in the Chałupy region (mean 65.7 ng g^−1^) (Table 4). The decline of Bivalvia in macrozoobenthos biomass was accompanied by an increase in the share of taxa with higher than average (in terms of bottom fauna) Hg_TOT_ concentrations (e.g. Oligochaeta, Gastropoda). As with the growing share of Crustacea in the area of Osłonino, this may lead to an increase in the load of Hg_TOT_ conveyed to higher trophic levels.

Changes in species composition and macroozoobenthos structure are also related to the emergence of new species, which most often reach the Baltic with the ballast water of ships (Leppakoski and Olenin 2000). Some non-indigenous species such as *M. arenaria* and *A. improvisus* were introduced into the Baltic Sea more than a hundred years ago, and their population in the basin having since stabilised has become a permanent feature of the macrofauna. However, more than half of the new species have been introduced into the Baltic post-1950, and their populations are now dynamically developing and colonising new areas. These include, among others, *R. harrisii* and *Gammarus tigrinius*, recorded in the Baltic Sea for the first time in 1951 and 1975, respectively. Another important species is the genus *Marenzelleria* sp., first observed in the Baltic Sea in the mid-1980s (Leppakoski and Olenin 2000; Janas and Kendzierska 2014).

In collected macrofauna, four non-native species were identified—*M. arenaria*, *Marenzelleria* sp., *R. harrisii* and *A. improvisus* (Table SII). The highest concentration of Hg among these non-indigenous species was measured in *Marenzelleria* sp., for which the average metal concentration was 63% higher than the mean Hg concentration in all analysed species and 41% higher than the mean Hg concentration measured in non-native species. The *Marenzelleria* genus was the latest of the non-native species to appear, which was first identified in the area of the Polish coast as recently as 1988 (Gruszka 1991). In the Puck Lagoon area, the highest density of *Marenzelleria* occurs at depths of over 4 m, but this species has also appeared sporadically in shallow areas in the past. In previous studies, no taxa were observed in the shallow part (< 1 m) of the coastal zone in Chałupy and Osłonino (Kotwicki 1997; Janas and Kendzierska 2014). In other shallow areas of the coastal zone, where the presence of *Marenzelleria* was detected at a depth of less than 1 m, the average abundance of the species between 1991 and 1992 was more than three times lower than in 2011–2013, while its average biomass was 10-fold less than reported by the authors of this work (Kotwicki 1997). This suggests that the *Marenzelleria* genus has settled into ever more shallow bottom areas, and the expansion of this species in the aquifer, as well as its individual traits, can significantly influence the transfer of Hg in the ecosystem. *Marenzelleria* penetrates into the sediment to a depth of 40 cm, thereby increasing the thickness of the populated sediment layer and the maximum depth of bioturbation. In addition, *Marenzelleria* is reported to migrate by active swimming and such movement may serve as an important link in energy transfer between pelagic and benthic subsystems (Bochert et al. 1996; Leppakoski and Olenin, 2000). As a result, *Marenzelleria* mobilises Hg-rich organic matter deposited in deeper sediment. In addition to this, it is characterised by rapid spread due to its high capacity for reproduction, rapid growth and longevity of life (as with the 2- to 3-year lifespan of Polychaeta) (Leppakoski and Olenin 2000), all of which facilitates the accumulation of Hg. The high concentration of Hg in the dynamically spreading *Marenzelleria* genus can also indirectly affect human health, particularly as this species is an important dietary component of flatfish and perch, both of which are caught for human consumption (Warzocha 2009).

## Summary

In the presented study, the effect of ecosystems’ characteristic on the bioaccumulation and biomagnification of Hg in marine the trophic chain was demonstrated. The previous works relating to this topic concerned mainly freshwater ecosystems (i.e. Campbell et al. 2005; Wyn et al. 2009; Kidd et al. 2012), while comparative studies for marine environment are still rare (Lavoie et al. 2013; Chouvelon et al. 2018). In addition, the previous research was usually conducted in systems characterised by higher productivity and biodiversity compared to the Baltic Sea.

Results of our ecosystem-comparative study showed that although the stations in the Puck Lagoon were situated in close proximity, the concentrations of Hg in the analysed benthic components, as well as its bioaccumulation and biomagnification in the trophic chain were different. An important factor shaping the level of Hg in investigated organisms was the inflow of allochthonous organic matter to the coastal waters. The increased inflow of terrigenous matter from land together with the shape of the coastline and sea bottom, which were conducive to the accumulation of organic matter near the shore, resulted in an increase in Hg_TOT_ concentration in suspended particulate matter and primary microproducers (epiphytes, phytoplankton). This led to an increase in the concentration of Hg_TOT_ in filter feeders. In the case of an area subjected to the inflow of marine organic matter, with Hg-rich fine organic particles coming from deeper regions of the bottom, an increase in Hg_TOT_ concentrations was observed in organisms feeding on sediment particles. Therefore, it is particularly important to take into account differences in the quality and quantity of organic matter at research stations.

An important factors contributing to the increase of the Hg_TOT_ load entering the trophic chain were environmental parameters. The physico-chemical characteristics of the environment, as well as its productivity, were found to be of particular importance in terms of the BSAF and BMF. Weaker uptake and trophic transfer of Hg_TOT_ was noted in the area with poorer oxygen conditions, lower pH and higher temperature of the near-bottom water. The introduction of Hg_TOT_ into the trophic chain may also be aggravated by the rebuilding of the macrofauna structure (increase in the biomass of species with high Hg_TOT_ concentration, with a simultaneous decrease of taxa with Hg_TOT_ level) and the expansion of non-native species. From the point of view of human health, the increase of Hg concentration in fish and invertebrates caught for consumption, one of the main routes by which Hg enters the body, is particularly dangerous. This is especially important for populations with a traditionally high dietary intake of fish and seafood, as they are particularly vulnerable to Hg poisoning.

## Electronic supplementary material

ESM 1 (PDF 518 kb)
