# Supplementary material for: The role of benthic macrofauna in the trophic transfer of mercury in a low-diversity temperate coastal ecosystem (Puck Lagoon, southern Baltic Sea)
Source: Environ Monit Assess. 2019 Feb 7;191(3):137. doi: 10.1007/s10661-019-7257-y (PMC6373316; doi:10.1007/s10661-019-7257-y)
Supplement: Supplementary file 1 — (PDF 518 kb) [file 10661_2019_7257_MOESM1_ESM.pdf]

Supplementary material

The supplementary data to the article *The role of benthic macrofauna in the trophic transfer of mercury in a low-diversity temperate coastal ecosystem (Puck Lagoon, southern Baltic Sea)* by A. Jędruch, M. Bełdowska and M. Ziółkowska (Institute of Oceanography, University of Gdańsk, Poland 🖂 Corresponding author: agnieszka.jedruch@ug.edu.pl)

Table SI Mean values of stable isotopes ratios of nitrogen and carbon ($\delta^{15}N$, $\delta^{13}C$) in various benthic components based on literature data

Ecosystem component			$\delta^{13}C$	$\delta^{15}N$	Region	Reference
SOM			-18.0	1.9	Puck Lagoon, Baltic Sea	Jankowska et al., 2016; 2018
POM (Osłonino)			-24.8	3.6	Puck Lagoon, Baltic Sea	Jędruch et al., 2017
POM (Chałupy)			-21.7	2.4	Puck Lagoon, Baltic Sea	Jędruch et al., 2017
detritus			-11.5	6.2	Puck Lagoon, Baltic Sea	Jankowska et al., 2016; 2018
phytoplankton			-21.5	4.9	northern Baltic Sea	Hansson et al., 1997
microphytobentos			-14.4	5.0	Bay of Biscay	Riera et al., 1999
epiphytes			-21.8	7.3	Puck Lagoon, Baltic Sea	Jankowska et al., 2016; 2018
macroalgae		*Enteromorpha* sp.	-19.2	5.8	Puck Lagoon, Baltic Sea	Sokołowski, 2009
		Chara sp.	-17.0	6.4	Puck Lagoon, Baltic Sea	Sokołowski, 2009
		P. littoralis	-21.7	7.6	Puck Lagoon, Baltic Sea	Jankowska et al., 2016; 2018
		Polysiphonia sp.	-25.0	8.1	Puck Lagoon, Baltic Sea	Jankowska et al., 2016; 2018
		Cladophora sp.	-22.8	8.2	Puck Lagoon, Baltic Sea	Jankowska et al., 2016; 2018
vascular plants		*M. spicatum*	-14.2	5.5	Puck Lagoon, Baltic Sea	Sokołowski, 2009
		P. pectinatus	-9.5	7.1	Puck Lagoon, Baltic Sea	Jankowska et al., 2016; 2018
		Z. marina	-10.0	8.3	Puck Lagoon, Baltic Sea	Jankowska et al., 2016; 2018
zooplankton			-21.7	8.5	northern Baltic Sea	Hansson et al., 1997
meiofauna			-19.8	6.1	Puck Lagoon, Baltic Sea	Jankowska, 2017
macrofauna	grazers	*T. fluviatilis*	-19.8	7.7	Puck Lagoon, Baltic Sea	Sokołowski, 2009
		Jaera sp.	-18.4	7.7	northern Baltic Sea	Kolb et al., 2010
		R. labiata	-20.3	8.1	Puck Lagoon, Baltic Sea	Sokołowski, 2009
		Peringia sp.	-19.7	8.9	Celtic Sea	Riera, 2010
		B. pilosa	-18.8	9.0	northern Baltic Sea	Nordström et al., 2009
		Idotea sp.	-18.0	9.5	Puck Lagoon, Baltic Sea	Sokołowski, 2009
		L. hookeri	-20.1	10.2	Balearic Sea	Prado et al., 2013
	suspensivore and/or detritivore	*Corophium* sp.	-21.3	8.2	Puck Lagoon, Baltic Sea	Sokołowski, 2009
		A. improvisus	-22.7	8.4	Puck Lagoon, Baltic Sea	Sokołowski, 2009
		C. glaucum	-22.0	8.7	Puck Lagoon, Baltic Sea	Sokołowski, 2009
		Oligochaeta	-23.4	8.7	Celtic Sea	Attrill et al., 2009
		L. balthica	-20.8	9.5	Puck Lagoon, Baltic Sea	Sokołowski, 2009
		M. arenaria	-22.2	9.6	Puck Lagoon, Baltic Sea	Sokołowski, 2009
	omnivorous	*Gammarus* sp.	-21.1	8.8	Puck Lagoon, Baltic Sea	Sokołowski, 2009
		Nemertea	-20.5	9.1	Waqouit Bay	Olsen et al., 2011
		R. harrisii	-20.4	9.5	Puck Lagoon, Baltic Sea	Sokołowski, 2009
		H. diversicolor	-21.0	10.3	Puck Lagoon, Baltic Sea	Sokołowski, 2009
		Marenzelleria sp.	-20.5	10.8	northern Baltic Sea	Karlson et al., 2015
		Insect larvae	-18.3	8.8	northern Baltic Sea	Nordstöm et al., 2009
	benthic fish	*P. pungitius*	-20.5	12.0	Gulf of Gdańsk, Baltic Sea	Sokołowski, 2009
		P. minutus	-19.0	12.4	Gulf of Gdańsk, Baltic Sea	Sokołowski, 2009
		P. flesus	-23.0	14.5	Gulf of Gdańsk, Baltic Sea	Sokołowski, 2009

SOM – sediment organic matter; POM – particulate organic matter

Table SII Species composition and structure of macrofauna communities in the coastal zone of the Puck Lagoon (southern Baltic Sea) in years 2011-2013

Classification	Osłonino										Chałupy									
	Abundace				Biomass				Frequency		Abundace				Biomass				Frequency	
	(indiv. m^{-2})		D		(g m^{-2})		D		C		(indiv. m^{-2})		D		(g m^{-2})		D		C	
	mean	range	(%)		mean	range	(%)		(%)		mean	range	(%)		mean	range	(%)		(%)	
**Bivalvia**																				
Cerastoderma glaucum (Bruguière, 1789)	45	1-80	0.2	Sr	53.75	1.28-125.09	2.8	Sd	43	As	167	6-453	2.9	Sd	2.03	0.09-5.73	4.3	Sd	93	E
Limecola balthica (Linnaeus, 1758)	55	3-227	1.0	R	62.58	2.08-189.12	6.4	D	100	E	–	–	–	–	–	–	–	–	–	–
Mya arenaria (Linnaeus, 1758)	23	1-53	0.1	Sr	21.28	17.28-25.28	0.5	Sr	57	C	13	13	0.1	Sr	2.92	2.54-3.30	3.1	Sd	14	Ad
**Crustacea**																				
Amphibalanus improvisus (Darwin, 1854)	–	–	–	–	–	–	–	–	–	–	13	13	0.0	Sr	0.69	0.69	0.4	Sr	7	Ad
Bathyporeia pilosa (Lindström, 1855)	13	13	0.1	Sr	1.41	1.41	0.0	Sr	14	Ad	–	–	–	–	–	–	–	–	–	–
Corophium sp. (Latreille, 1806)	3038	6-19227	31.0	E	236.8	2.56-757.92	30.4	E	79	E	198	13-933	2.1	Sd	0.71	0.02-3.66	2.6	Sd	57	C
Gammarus sp. (Fabricius, 1775)	183	13-680	1.9	R	49.47	0.78-179.36	7.0	D	93	E	191	13-893	3.4	Sd	1.47	0.03-6.15	9.3	D	93	E
Idotea sp. (Fabricius, 1798)	148	6-400	0.8	Sr	32.36	2.85-124.54	2.9	Sd	50	As	323	27-787	4.4	Sd	1.47	0.07-3.32	7.8	D	71	C
Jaera sp. (Leach, 1814)	–	–	–	–	–	–	–	–	–	–	27	27	0.1	Sr	0.02	0.02	0.0	Sr	14	Ad
Rhithropanopeus harrisii (Gould, 1841)	13	13	0.1	Sr	30.08	30.08	0.4	Sr	21	Ad	7	7	0.0	Sr	4.52	4.52	2.4	Sd	21	Ac
Lekanesphaera hookeri (Leach, 1814)	–	–	–	–	–	–	–	–	–	–	125	3-640	2.0	Sd	0.74	0.74	3.9	Sd	86	E
**Gastropoda**																				
Peringia sp. (Hartmann, 1821)	3711	61-9627	38.0	E	64.01	25.58-109.63	9.8	D	93	E	2869	222-7880	50.1	E	2.75	0.58-8.37	17.5	E	93	E
Radix labiata (Rossmässler, 1835)	–	–	–	–	–	–	–	–	–	–	38	13-80	0.2	Sr	0.7	0.53-0.87	1.5	R	43	As
Theodoxus fluviatilis (Linnaeus, 1758)	–	–	–	–	–	–	–	–	–	–	125	6-360	1.7	R	0.84	0.15-2.51	4.0	Sd	71	C
**Polychaeta**																				
Hediste diversicolor (Müller, 1776)	616	60-1853	5.3	D	220.51	26.99-1063.52	31.1	E	79	E	391	10-1173	7.4	D	5.67	0.88-23.26	33.1	E	100	E
Marenzelleria sp. (Mesnil, 1896)	71	13-160	0.3	Sr	14.06	1.31-40.03	1.1	R	43	As	18	18	0.1	Sr	0.08	0.08	0.1	Sr	14	Ad
Streblospio shrubsolii (Buchanan, 1890)	13	13	0.1	Sr	1.92	1.92	0.0	Sr	21	Ac	–	–	–	–	–	–	–	–	–	–
**Oligochaeta**	1299	9-6387	12.2	E	45.57	1.12-208.64	6.4	D	86	E	1199	55-3187	21.1	E	0.69	0.06-2.16	4.4	Sd	93	E
**Nemertea**	13	13	0.0	Sr	1.44	1.44	0.0	Sr	14	Ad	28	13-93	0.4	Sr	0.33	0.04-0.55	1.0	R	57	C
**Insect larvae**	470	1-7413	8.9	D	11.26	2.19-43.36	1.2	R	71	C	326	13-1320	4.0	Sd	1.07	0.03-6.64	4.6	Sd	64	C

D – domination factor (Eq. 1 in the article); E – eudominant, D – dominant, Sd – subdominant, R – recedent, Sr - subrecedent
C – frequency or occurrence (Eq. 2 in the article); E – euconstant, C – constant, As – accessory, Ad – accidental

Table SIII The Spearman's correlations coefficients between selected species of macrozoobenthos and sources of organic matter of the coastal zone of the Puck Lagoon (southern Baltic Sea) in years 2011-2013, as an indicators of the feeding mode/habit

Station	Taxa	Trophic group	POM	SOM	FLSM	phyto-plankton	epiphyton	macro-algae	vascular plants
Osłonino	*C. glaucum*	suspensivore	0.43			0.67			
	Corophium sp.	suspensivore/ detritivore	0.52	0.53			0.67		
	H. diversicolor	omnivore		0.52			0.44		
Chałupy	*M. arenaria*	suspensivore		0.81	0.62				
	Idotea sp.	grazer		0.67				0.46	0.68
	Peringia sp.	grazer		0.56					

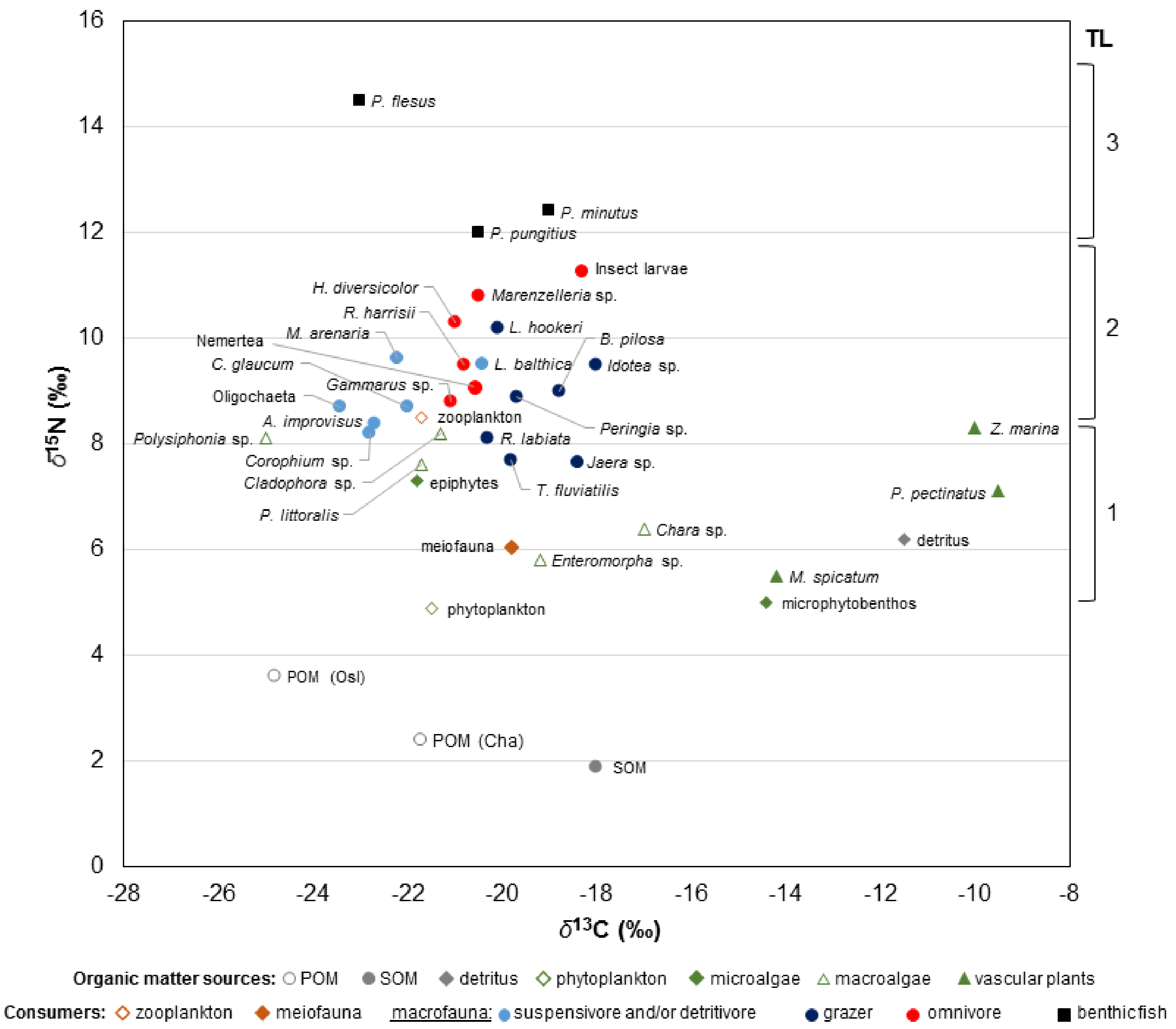

Figure SI Relationship between mean values of stable isotopes ratios of nitrogen and carbon (δ^{15}N, δ^{13}C) and the trophic level (TL) in various benthic components of the coastal zone (Osłonino and Chałupy stations) of the Puck Lagoon in years 2011-2013 (data sources can be found in Table SI)
